# Identification of novel pathogenic roles of BLZF1/ATF6 in tumorigenesis of gastrointestinal stromal tumor showing Golgi-localized mutant KIT

**DOI:** 10.1038/s41418-023-01220-2

**Published:** 2023-09-13

**Authors:** Yujin Kwon, Jiyoon Kim, Su-Yeon Cho, Yoon Jin Kang, Jongsoo Lee, Jaeyoung Kwon, Hyungjin Rhee, Sebastian Bauer, Hyung-Sik Kim, Esak Lee, Han Sang Kim, Jae Hung Jung, Hoguen Kim, Won Kyu Kim

**Affiliations:** 1 Natural Product Research Center, Korea Institute of Science and Technology (KIST), Gangneung, 25451 South Korea; 2 Division of Bio-Medical Science & Technology, University of Science and Technology (UST), Daejeon, 34113 South Korea; 3 Department of Pharmacology, Department of Biomedicine & Health Sciences, College of Medicine, The Catholic University of Korea, Seoul, 06591 South Korea; 4 Department of Marine Life Sciences, College of Life Science, Gangneung-Wonju National University, Gangneung, 25457 South Korea; 5 Department of Urology, Urologic Science Institute, Yonsei University College of Medicine, Seoul, 03722 South Korea; 6 Natural Product Informatics Research Center, Korea Institute of Science and Technology (KIST), Gangneung, 25451 South Korea; 7 Department of Radiology, Research Institute of Radiological Science, Center for Clinical Imaging Data Science, Severance Hospital, Yonsei University College of Medicine, Seoul, 03722 South Korea; 8 Sarcoma Center, West German Cancer Center, University Hospital Essen, University Duisburg-Essen, Germany and German Cancer Consortium (DKTK), Essen, 45141 Germany; 9 Department of Oral Biochemistry; Dental and Life Science Institute, School of Dentistry, Pusan National University, Yangsan, 50612 South Korea; 10 Nancy E. and Peter C. Meinig School of Biomedical Engineering, Cornell University, Ithaca, NY 14853 USA; 11 Division of Medical Oncology, Department of Internal Medicine, Yonsei Cancer Center, Yonsei University College of Medicine, Seoul, 03722 South Korea; 12 Department of Urology, Yonsei University Wonju College of Medicine/Center of Evidence Based Medicine Institute of Convergence Science, Wonju, 26426 South Korea; 13 Department of Pathology, Yonsei University College of Medicine, Seoul, 03722 South Korea; 14 Department of Convergence Medicine, Yonsei University Wonju College of Medicine, Wonju, 26426 South Korea

**Keywords:** Sarcoma, Chaperones

## Abstract

Gastrointestinal stromal tumors (GISTs) frequently show KIT mutations, accompanied by overexpression and aberrant localization of mutant KIT (MT-KIT). As previously established by multiple studies, including ours, we confirmed that MT-KIT initiates downstream signaling in the Golgi complex. Basic leucine zipper nuclear factor 1 (BLZF1) was identified as a novel MT-KIT-binding partner that tethers MT-KIT to the Golgi complex. Sustained activation of activated transcription factor 6 (ATF6), which belongs to the unfolded protein response (UPR) family, alleviates endoplasmic reticulum (ER) stress by upregulating chaperone expression, including heat shock protein 90 (HSP90), which assists in MT-KIT folding. BLZF1 knockdown and ATF6 inhibition suppressed both imatinib-sensitive and -resistant GIST in vitro. ATF6 inhibitors further showed potent antitumor effects in GIST xenografts, and the effect was enhanced with ER stress-inducing drugs. ATF6 activation was frequently observed in 67% of patients with GIST (*n* = 42), and was significantly associated with poorer relapse-free survival (*P* = 0.033). Overall, GIST bypasses ER quality control (QC) and ER stress-mediated cell death via UPR activation and uses the QC-free Golgi to initiate signaling.

## Introduction

Gastrointestinal stromal tumor (GIST), the most common mesenchymal tumor in the stomach and small intestine, is a well-known model of oncogene addiction. Approximately 75% of patients with GIST show primary gain-of-function mutations in *KIT*, and secondary mutations additionally occur in *KIT* during imatinib treatment [1]. The primary mutations are mostly detected in exon 11 of *KIT*, and patients with these mutations show more than 80% of the overall response rate (ORR) to imatinib [2]. The major challenge of GIST treatment is that there are insufficient regimens for patients showing poor response or developing resistance to imatinib [3]. Other kinase inhibitors, such as sunitinib and regorafenib, and more recently ripretinib, have been applied to patients who are refractory to imatinib. However, these drugs have shown limited therapeutic efficacy, especially in a metastatic setting [4]. Therefore, breakthroughs beyond *KIT*, such as identification of novel druggable targets and tumorigenic mechanisms, are needed for GIST treatment.

Mutant KIT (MT-KIT) was previously reported to be aberrantly localized in the Golgi complex and to exhibit sustained activation [5]. However, it remains unclear how MT-KIT escapes the endoplasmic reticulum quality control (ERQC) system to reach the Golgi complex, and how the Golgi-retained MT-KIT contributes to GIST pathogenesis. Therefore, identifying the mechanisms underlying these processes could provide important therapeutic insights into the challenges faced by conventional GIST treatment.

Cancer cells are constantly exposed to extrinsic and intrinsic stresses, such as hypoxia, nutrition insufficiency, and increased folding burden of proteins, which collectively cause ER stress and cell death. To survive unfavorable conditions, cancer cells activate the unfolded protein response (UPR) pathway, which ameliorates ER stresses by regulating protein degradation, chaperone expression, and adaptive pathways related to cell proliferation and survival [6]. GIST is a relatively large solid tumor and most solid tumors develop regions of low oxygen tension. In addition, GIST exhibits MT-KIT folding stress [7]. Therefore, GIST is subjected to chronic ER stress during tumorigenesis, which suggests the potential role of UPR in overcoming ER stress. Moreover, UPR is involved in mutant protein folding, which also indicates that UPR might be involved in ERQC bypass and Golgi retention of MT-KIT.

In this study, we aimed to elucidate the pathological mechanisms of Golgi-retained MT-KIT in GISTs to better understand its biological and clinical significance, and to investigate the unknown roles of ER stress and UPR to discover potential non-KIT drug targets. Here, we show the underlying mechanism for the tumorigenic roles of Golgi-localized MT-KIT, with the assistance of BLZF1 and a novel pro-survival mechanism, based on constant activation of the ATF6-dependent UPR pathway in GIST.

## Results

### MT-KIT is primarily localized in the Golgi complex in GIST cell lines and tissues

**Fig. 1:**
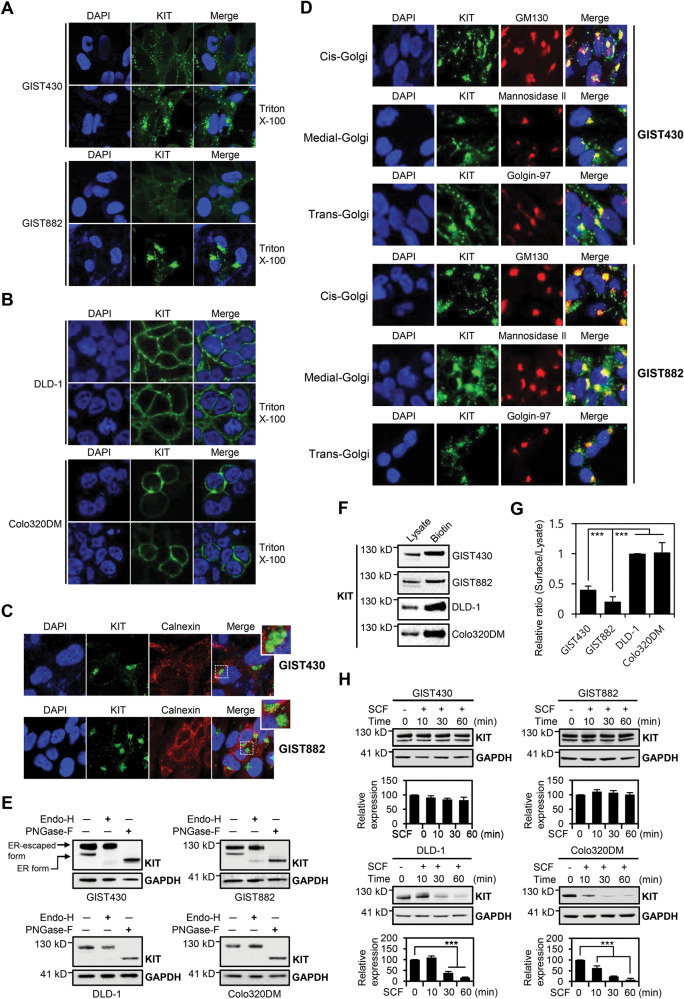
Mutant KIT (MT-KIT) and wild type KIT (WT-KIT) show perinuclear Golgi and membranous expression patterns, respectively. **A**, **B** Immunocytochemistry (ICC) analysis of KIT expression in gastrointestinal stromal tumor (GIST) (GIST430 and GIST882) and colon cancer (CC) cells (DLD-1 and Colo320DM) with or without permeabilization using triton X-100. **C** ICC analysis of KIT and calnexin (endoplasmic reticulum (ER) marker) in GIST cells. **D** ICC analysis of KIT, GM130 (cis-Golgi), mannosidase ll (medial-Golgi), and Golgin-97 (trans-Golgi) in GIST cells. **E** Lysates from GIST and CC cells were deglycosylated using endoglycosidase H (Endo-H) or PNGase-F, and analyzed by western blotting. **F** Biotinylation, streptavidin pull-down, and western blotting were performed using GIST and CC cells. Because the surface expression of MT-KIT was low, twice the amount of biotin-labeled lysates was used for GIST cells than in CC cell lysates. **G** Quantification of the band intensities for the western blots as shown in **F**. **H** Stem cell factor (SCF)-treatment-mediated KIT degradation was measured by western blot lysates in GIST and CC cells. Band intensities of the western blots were quantified. One-way ANOVA with a post-hoc test was performed to compare multiple means (*p < 0.05, **p < 0.01, ***p < 0.001). Error bars in **G** and **H** represent the standard deviation (SD) of the mean of three independent western blots.

To detect the cellular localization of MT-KIT (GIST cells: GIST430 and GIST882) and wild-type KIT (colon cancer cells: DLD-1 and Colo320DM), immunocytochemistry (ICC) analysis was performed. In GIST cells, fluorescence signal of MT-KIT was barely observed at the plasma membrane (PM) without permeabilization, whereas a strong perinuclear fluorescence signal of MT-KIT was detected following permeabilization (Fig. 1A). In colon cancer (CC) cells, wild-type KIT (WT-KIT) was detected only in the PM, regardless of permeabilization (Fig. 1B). The perinuclear MT-KIT did not colocalize with the ER marker calnexin, but clearly colocalized with the cis- (GM130), medial- (mannosidase ll), and trans-Golgi (Golgin-97) markers (Fig. 1C, D). Quantification of immunofluorescence intensity further demonstrated that MT-KIT evidently colocalized to the Golgi complex (Supplementary Fig. S1). Deglycosylation analysis of MT- and WT-KIT further revealed that KIT localized in the ER (endoglycosidase H-sensitive) was barely detected in GIST and CC cells, which supports the hypothesis that MT-KIT in GISTs is primarily localized in post-ER organelles (Fig. 1E). Golgi localization of MT-KIT was validated in tissues from 42 patients with GIST. Immunohistochemistry (IHC) analysis showed that perinuclear MT-KIT expression was detected in 28 patients (67%) (Supplementary Fig. S2 and Supplementary Table S1).

Although ICC analysis showed Golgi localization of MT-KIT, membranous MT-KIT expression in GISTs and CCs was quantitatively measured by biotin labeling and western blot analysis of PM proteins. Since the membranous MT-KIT expression in GIST cells was too low, the amount of GIST whole-cell lysate used for biotin-labeled protein pulldown was doubled than that in CCs. The ratios between membranous KIT levels compared with whole-cell lysates were approximately 0.40 and 0.20 for GIST430 and GIST882 cells, respectively, while those for CC cells were approximately 1.0 (Fig. 1F, G). The surface expression of MT-KIT in GIST430 cells was higher than in GIST882 cells due to the heterozygous mutation in GIST430 cells [8].

### Membranous MT-KIT is not involved in GIST tumorigenesis

Despite a small fraction of the total MT-KIT, weak membranous KIT expression was detected in GIST cells (Fig. 1F). Therefore, a ligand tolerance assay was performed to test if the membranous MT-KIT responds to its ligand, the stem cell factor (SCF). As previously reported [9], SCF treatment rapidly downregulated WT-KIT expression in CC cells. However, MT-KIT levels barely changed in GIST cells following SCF treatment (Fig. 1H). The fate of membranous MT-KIT was tracked in a time-dependent manner to understand its role in GIST tumorigenesis. GIST882 cells harboring the homozygous *KIT* mutation were used for analysis. GIST882 cells were labeled with a fluorescent-dye-conjugated KIT antibody and examined over time using a confocal microscope. Membranous MT-KIT was internalized within 15 min and disappeared within 1 h. Colocalization was not observed between MT-KIT and a Golgi complex marker, GM130, until MT-KIT disappeared, which excludes the possibility that Golgi retention of MT-KIT is mediated by the endosome-to-trans-Golgi retrieval pathway, a reported Golgi retention mechanism of the G-protein-coupled receptor [10] (Supplementary Fig. S3A). Next, the involvement of PM quality-control (PMQC) in the disappearance of MT-KIT was investigated. The labeling experiment was repeated to evaluate the colocalization of MT-KIT with an early endosome marker, EEA1, or a lysosomal marker, LAMP1, involved in the PMQC processes [11]. Colocalization of membranous MT-KIT with EEA1 and LAMP1 was clearly observed 15 and 30 min after tracking, and disappeared after 1 h (Supplementary Fig. S3B, C).

To quantitatively validate the ICC results, western blot analysis of membranous KIT was performed in GISTs and CCs. In GISTs, the remaining membranous MT-KIT levels was measured through biotin-labeled protein pulldown assay and western blot at the indicated time-points. Bafilomycin A1 (BafA1), a lysosomal inhibitor, was used to validate lysosome-mediated protein degradation. CCs were included as controls to determine the original stability of WT-KIT. Since WT-KIT is mostly expressed in the PM, the remaining WT-KIT abundance was measured at the indicated time-points after cycloheximide (CHX) treatment. After 4 h, >50% of the original WT-KIT level remained (Supplementary Fig. S4A), whereas in GIST882 cells, 8% remained. MT-KIT degradation was efficiently blocked by BafA1 treatment (Supplementary Fig. S4B). These findings suggest that membranous MT-KIT is hardly involved in GIST tumorigenesis due to its low membranous protein level caused by Golgi retention and plasma membrane quality control (PMQC).

### Neither PM nor ER is the site for MT-KIT downstream signaling activation

**Fig. 2:**
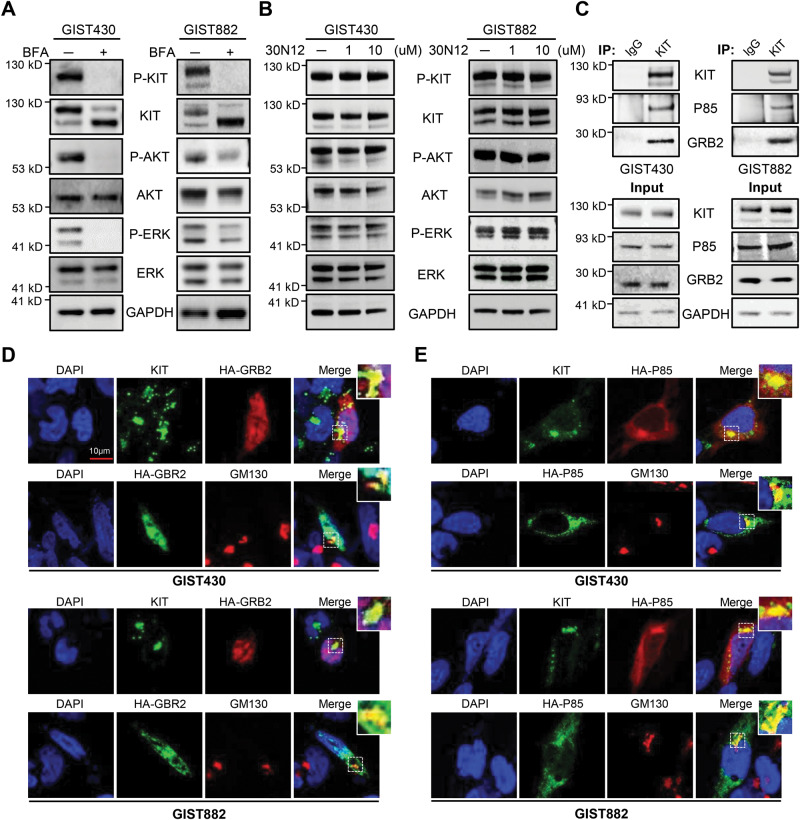
Golgi complex is the main site where MT-KIT initiates downstream oncogenic signaling. **A** The status of downstream effector molecules in the KIT signaling pathway was analyzed by western blotting GIST cells with or without treatment with Brefeldin A (BFA, 5 µg/mL) for 4 h, a compound that blocks ER to Golgi trafficking. **B** The same analysis was performed as in **A** using GIST cells with or without treatment for 18 h with 1 or 10 µM 30N12, a compound that blocks trans-Golgi trafficking to the plasma membrane (PM). **C** Immunoprecipitation of GIST430 and GIST882 cell lysates was performed to evaluate the interaction of MT-KIT with P85 and GRB2, the most upstream molecules of the PI3K/AKT pathway and MAPK/ERK pathway, respectively. **D**, **E** Confocal microscopic analysis of KIT, GRB2, P85, and GM130 in GIST cells transfected with HA-GRB2 or HA-P85 expression vectors was performed. HA-GRB2 and HA-P85 were labeled with hemagglutinin (HA).

Because of low protein levels and rapid degradation of membranous MT-KIT, we hypothesized that the Golgi-localized MT-KIT is the primary contributor to sustained activation of downstream signaling in GISTs. We first aimed to exclude the possibility that ER- or PM-localized MT-KIT initiates downstream signaling. Brefeldin A (BFA) was used to block ER-to-Golgi trafficking, thereby inducing ER retention of MT-KIT. ICC analysis showed that BFA led to ER retention of MT-KIT (Supplementary Fig. S5). Western blot analysis showed that BFA dramatically reduced phospho-KIT, phospho-AKT, and phospho-ERK levels, indicating that ER-retained MT-KIT was insufficient to activate downstream signaling (Fig. 2A). On the other hand, the levels of phospho-AKT and phospho-ERK were minimally affected by BFA treatment in CC cells, whereas KIT was retained in the ER (Supplementary Fig. S6A). The involvement of membranous MT-KIT in downstream signaling was also investigated using 30N12, an inhibitor of trans-Golgi-to-PM trafficking [12]. To validate the effects of 30N12, western blot and ICC analysis of WT-KIT was performed in SCF-treated CC cells treated with or without 30N12. Treatment with 30N12 efficiently induced WT-KIT retention in the Golgi complex, and reduced phospho-KIT, phospho-AKT, and phospho-ERK levels, that were upregulated by SCF treatment (Supplementary Fig. S6B, C). However, in GIST cells, 30N12 treatment did not change the phosphorylation levels of effector molecules, indicating that PM-localized MT-KIT was also insufficient to activate downstream signaling (Fig. 2B).

### The Golgi complex, where MT-KIT is predominantly localized, is the site through which MT-KIT delivers downstream oncogenic signaling in GIST

To investigate whether Golgi-retained MT-KIT primarily contributes to downstream oncogenic signaling activation, we investigated the interaction of MT-KIT with P85 and GRB2, the upstream effectors of the PI3K/AKT and MAPK/ERK pathways, respectively. Immunoprecipitation assays were performed using GIST cell lysates incubated with IgG (control) or KIT antibody. This analysis showed that the endogenous MT-KIT bound to both P85 and GRB2 (Fig. 2C). The interaction of MT-KIT with GRB2 and P85 was further verified through ICC analysis. Since there are no commercial antibodies for ICC analysis of P85 and GRB2, HA-tagged P85 and GRB2 expression vectors were constructed. After vector transfection into GIST cells, cellular localization of MT-KIT, HA-P85, and HA-GRB2 were examined. GRB2 and P85 clearly showed colocalization with MT-KIT and GM130 (Fig. 2D, E). Quantification of immunofluorescence intensity further demonstrated that GRB2 and P85 colocalize with MT-KIT and GM130 at the Golgi complex (Supplementary Fig. S7). The localization of GRB2 and P85 after SCF treatment in CC cells was further investigated to compare the PI3K/AKT and MAPK/ERK pathway activation mediated by WT-KIT and MT-KIT. In contrast to GIST cells, ICC analysis showed that SCF treatment recruited P85 and GRB2 to the peri-PM region, where activated WT-KIT was localized (Supplementary Fig. S8). These data showed that MT-KIT directly initiates downstream signals from the Golgi complex, whereas WT-KIT initiates signals from the PM.

### BLZF1, a Golgi-resident protein, is required for MT-KIT expression in the Golgi complex

**Fig. 3:**
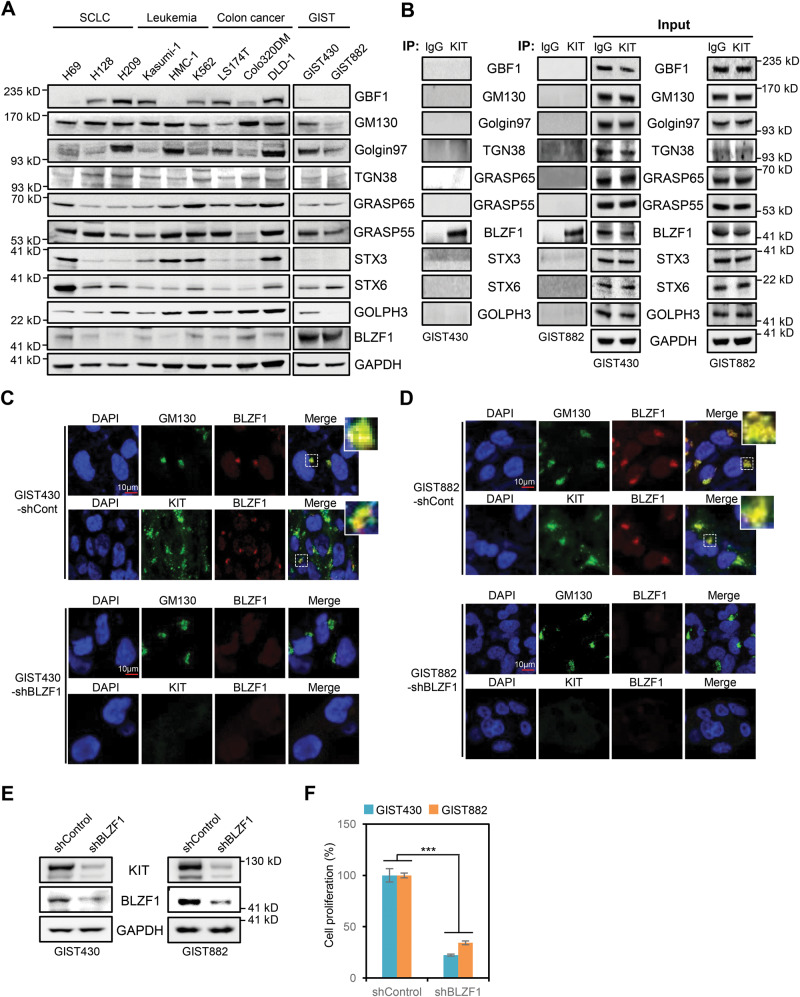
BLZF1 is a novel KIT binding partner, which is indispensable for tethering MT-KIT in the Golgi complex. **A** Western botting was performed using antibodies against ten Golgi-related proteins in small cell lung carcinoma (SCLC), leukemia, CC, and GIST cell lines expressing WT-KIT or MT-KIT. **B** Immunoprecipitation analysis was performed in GIST cells using a KIT antibody and binding between KIT and Golgi-related proteins was evaluated by western blotting. **C**, **D** Confocal microscopic analysis of GM130, BLZF1, and KIT in GIST cells with or without BLZF1 shRNA treatment. **E** Western blotting analysis of KIT and BLZF1 in GIST cells with or without BLZF1 shRNA treatment. **F** A proliferation assay was performed in GIST cells with or without BLZF1 shRNA treatment. Cell growth was measured by MTT analysis 72 h after BLZF1 shRNA treatment. Error bars in **F** represents the SD of the mean of three independent experiments. One-way ANOVA with a post-hoc test was performed to compare multiple means (*p < 0.05, **p < 0.01, ***p < 0.001).

Since the Golgi complex is a hub for constant protein trafficking, a complementary mechanism is necessary for the retention of MT-KIT. Therefore, we aimed to identify a possible MT-KIT-binding partner from the Golgi-related proteins that could tether MT-KIT to the Golgi complex during trafficking. Since there are only a few reported Golgi-related proteins [13–15], all possible candidates with commercially available antibodies were selected. Western blot was performed on ten Golgi proteins (GBF1, GM130, Golgin97, TGN38, GRASP65, GRASP55, BLZF1, STX3, STX6, and GOLPH3) in small-cell lung cancer (SCLC), leukemia, colon cancer, and GIST cell lines expressing WT-KIT or MT-KIT. All Golgi proteins showed variable levels in the cell lines, except for BLZF1 that selectively showed high protein levels in GIST 430 and GIST 882 cells (Fig. 3A). BLZF1 expression was also similarly high in imatinib-resistant GIST430-V654A and GIST48 cells (Supplementary Fig. S9). Moreover, immunoprecipitation with KIT antibody revealed that MT-KIT specifically bound to BLZF1 (Fig. 3B). The colocalization of MT-KIT and BLZF1 in the Golgi complex of GIST cells was validated by ICC analysis (Fig. 3C, D). On the other hand, fluorescence signal of BLZF was barely detected in the Golgi complex of CC and leukemia cell lines (DLD-1 and HMC-1) (Supplementary Fig. S10). BLZF1 knockdown with short hairpin RNA (shRNA) dramatically downregulated MT-KIT expression, as demonstrated by ICC and western blot analyses (Fig. 3C–E). BLZF1 knockdown strongly inhibited cell growth in both imatinib-sensitive GIST430 and GIST 882 cells, and in imatinib-resistant GIST430-V654A and GIST48 cells (Fig. 3F and Supplementary Fig. S11). These findings collectively suggest that BLZF1 is required for Golgi retention and stable expression of MT-KIT, and consequently for GIST cell growth.

### ATF6, a pro-survival ER stress sensor, is constantly activated in GISTs

**Fig. 4:**
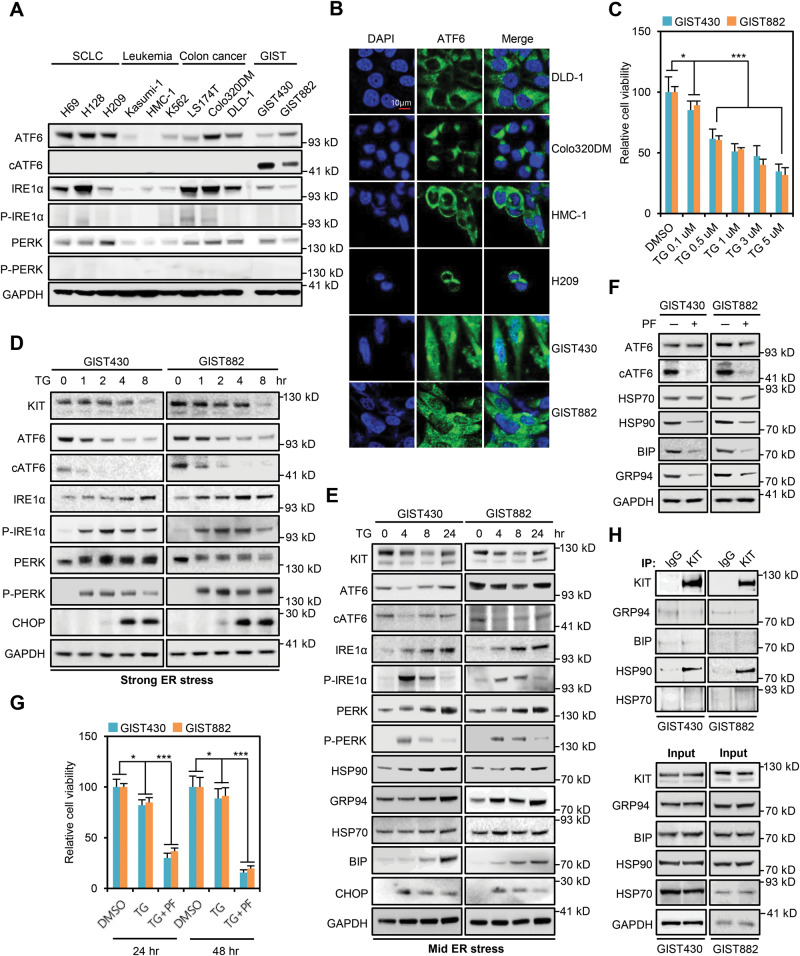
GISTs intrinsically adapt to ER stress in an ATF6-dependent manner, which is beneficial for folding and overexpression of MT-KIT. **A** Activation status of the three arms of the unfolded protein response (UPR) pathway (ATF6, IRE1α, and PERK) was measured by western blotting in a panel of cancer cell lines expressing WT-KIT or MT-KIT. **B** Confocal microscopy was performed to evaluate the nuclear localization of ATF6 in various cancer cell lines selected from the cell line panel used in A. **C** Cell viability was measured in GIST cells treated with various doses (0.1–5 µM) of an ER-stress inducer, thapsigargin (TG) for 24 h. **D** GIST cells were treated with 5 µM TG (strong ER-stressor) across various timepoints, and the expression of the three arms of the UPR pathway, a cell-death marker (CHOP), and KIT was measured by western blotting. **E** GIST cells were treated with 0.1 µM TG (mild ER-stressor) across various timepoints, and the expression of the markers measured in D and chaperones (HSP70, HSP90, BIP, and GRP94) was measured by western blotting. **F** Expression of ATF6 and chaperones was measured by western blotting after treatment with 30 µM PF429242 (PF), which blocks ATF6 cleavage. **G** Cell viability of GIST cells treated with 0.1 µM TG and PF was measured after 24 and 48 h. **H** Immunoprecipitation of GIST cell lysates was performed with a control IgG or KIT antibody, and western blotting was performed against chaperones. Error bars in **C** and **G** represent the SD of the mean of three independent experiments. One-way ANOVA with a post-hoc test was performed to compare multiple means (*p < 0.05, **p < 0.01, ***p < 0.001).

It remains unclear how MT-KIT bypasses ERQC to reach the Golgi complex. As shown in Fig. 1E, MT-KIT in GISTs was primarily in the post-ER form, which indicates that MT-KIT is folded enough to avoid ER accumulation and stress induction. Since GIST cells are constantly exposed to nutrient deficiency and hypoxia that cause ER stress. Therefore, we hypothesized that GISTs might have acquired a UPR-related intrinsic mechanism to relieve ER stress during tumorigenesis. The activation status of the three sensors of the UPR pathways (ATF6, IRE1α, and PERK) was measured in the cell line panel shown in Fig. 4A. Western blot analysis showed that GIST cells exclusively and constitutively expressed the cleaved form of ATF6 (cATF6), which is a transcriptional activator that upregulates the expression of chaperones and cell survival-related genes [6] (Fig. 4A). ICC analysis further showed that cATF6 was localized in the nucleus of GIST cells, whereas no nuclear fluorescence signal was detected in other cancer cells (Fig. 4B). These results suggest that GISTs may take advantage of ATF6 activation as a survival strategy against various cellular stressors.

### Sustained activation of ATF6 is a novel mechanism for GIST cell survival

To investigate ATF6 involvement in the intrinsic tolerance of GISTs to ER stress, the GIST cell viability was measured 24 h after treatment with an ER stress inducer thapsigargin (TG). GIST cells were treated with various concentrations of TG commonly used in previous studies [16, 17]. TG treatment decreased GIST cell viability in a dose-dependent manner (0.1–5 µM) (Fig. 4C). Based on the results, mild (0.1 µM TG) and strong (5 µM TG) ER stress-inducing conditions were selected. Because of drastic cell death after 24 h of treatment with 5 µM TG, the activation status of ATF6, IRE1α, and PERK was measured for up to 8 h. The expression of CHOP (a cell death-related marker) and MT-KIT was also measured. Western blot analysis showed that strong ER stress rapidly and drastically reduced ATF6, cleaved-ATF6 (cATF6), and MT-KIT levels over time, whereas phospho-IRE1α, phospho-PERK, and CHOP levels drastically increased after 1 h of treatment with TG and continuously increased for 8 h (Fig. 4D). In the case of mild ER stress, ER stress-adaptive chaperones (BIP, GRP94, HSP70, and HSP90) were analyzed for up to 24 h. ATF6, cATF6, and MT-KIT levels showed a moderate decrease until 8 h of treatment with TG but were fully restored after 24 h of TG treatment. On the other hand, phospho-IRE1α, phospho-PERK, and CHOP levels increased until 8 h of TG treatment, but were reduced to basal levels after 24 h. HSP90, BIP, and GRP94 levels gradually increased up to 24 h of TG treatment, while CHOP levels showed a gradual decrease after it peaked at 4 h of post-treatment (Fig. 4E). To validate the direct involvement of ATF6 pathway in mild ER stress adaptation of GISTs, an ATF6 inhibitor, PF429242 (PF), was used to block ATF6 cleavage. PF was used at a concentration of 30 µM commonly used in previous in vitro and in vivo studies [16, 18]. Western blot analysis showed that PF effectively inhibited ATF6 cleavage, and was accompanied by a drastic decrease in HSP90, BIP, and GRP94 levels (Fig. 4F). Under PF-mediated ATF6 inhibition, even mild TG resulted in a dramatic cell death (Fig. 4G). The upregulation of HSP90, BIP, and GRP94 was significantly delayed in GIST cells treated with both PF and mild TG. The upregulation of CHOP remained even after 24 h of TG and PF treatment (Supplementary Fig. S12). Next, the relationship between sustained ATF6 activation and folding and overexpression of MT-KIT was investigated to analyze the interaction between the MT-KIT and chaperones. Immunoprecipitation analysis showed that MT-KIT specifically bound to HSP90 (Fig. 4H). These results suggest that ATF6 plays a pro-survival role against ER stress-mediated cell death, which also benefits MT-KIT folding.

### Pharmacological inhibition of ATF6 perturbs GIST cell growth irrespective of imatinib resistance and shows enhanced anti-tumor effects when combined with ER stress-inducing drugs

**Fig. 5:**
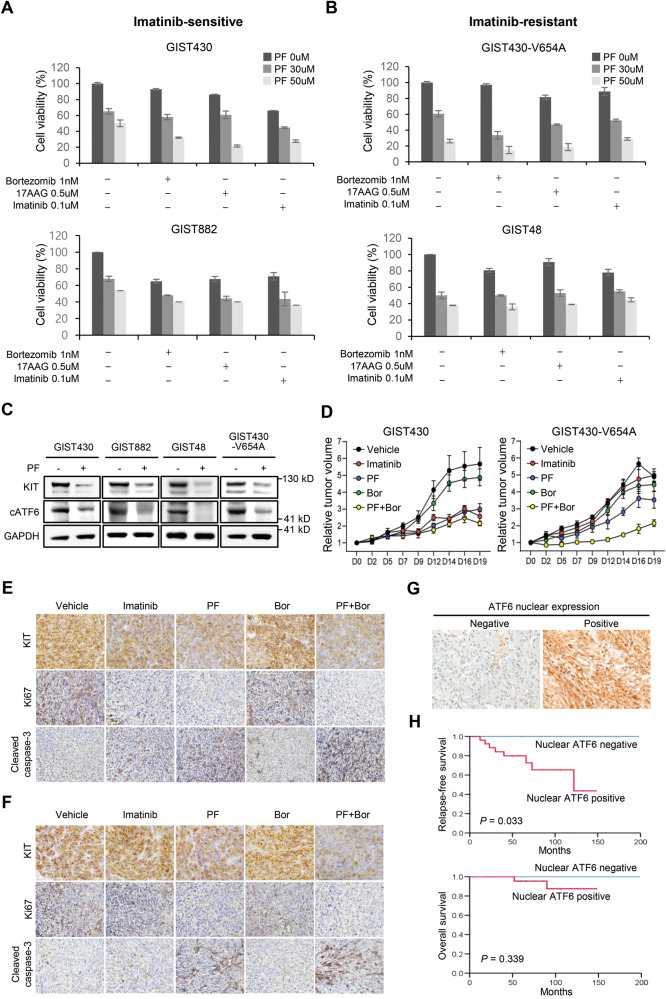
Synergic anti-cancer effect of ER stress inducer and ATF6 inhibitor combination treatment on GIST growth, and the prognostic significance of ATF6 nuclear expression in patients with GIST. **A**, **B** Cell viability after 72 h of treatment with PF429242 alone (PF, 30 µM and 50 µM) and PF with ER stress-inducing drugs (0.5 µM 17AAG and 1 nM bortezomib) was analyzed in imatinib-sensitive GIST430 and GIST882 cells, and imatinib-resistant GIST48 and GIST430-V654A cells. Imatinib (IM, 0.1 uM) was used as a control drug and the combined effect with PF was evaluated. **C** The expression of KIT and cATF6 was measured by western blotting after treatment of PF (30 µM). **D** The anti-tumor effect was measured in a GIST430 xenograft mouse model and GIST430-V654A model. Relative tumor volume was measured at indicated days in vehicle, IM (50 mg/kg), PF (30 mg/kg), bortezomib (Bor, 1 mg/kg), and PF with bortezomib treatment groups. Each group consisted of 5 mice. **E**, **F** Immunohistochemistry (IHC) analysis of KIT, Ki67, and cleaved caspase-3 was performed using tumor tissues obtained from xenograft mouse models. **G** Nuclear expression of ATF6 was evaluated by IHC analysis in 42 GIST tissues. Patients were divided into nuclear ATF6 expression negative and positive groups. **H** Relapse-free and overall survival curves were plotted by Kaplan–Meier analysis and assessed by log-rank test, according to nuclear ATF6 expression. Error bars represent the SD of the mean of three independent experiments. One-way ANOVA with a post-hoc test was performed to compare multiple means (*p < 0.05, **p < 0.01, ***p < 0.001).

To verify the significance of ATF6 as a therapeutic target in GISTs, the antitumor effects of PF, Ceapin-A7, and melatonin, which are reported ATF6 inhibitors, were investigated. The efficacy of these inhibitors in combination with ER stress-inducing drugs, such as bortezomib and 17AAG, were further evaluated. To mimic a constant ER stress condition in vivo, analysis was performed with or without TG, and imatinib was used as a control drug. Cell viability analysis showed that ATF6 inhibitors alone efficiently suppressed GIST cell growth, irrespective of imatinib resistance, while the antitumor effect was synergistically enhanced when the ATF6 inhibitors are combined with ER stress-inducing drugs. The synergistic antitumor effect was also observed with imatinib (Fig. 5A, B, and Supplementary Fig. S13). The synergistic effects between ATF6 inhibition and other drugs (bortezomib, 17AAG, and imatinib) were assessed using the highest single agent (HSA) score analysis [19]. The results of the calculations revealed significant synergistic effects (HSA score >10) when ATF6 inhibition with PF429242 was combined with any of the drugs (Supplementary Fig. S14).

Treatment with each ATF6 inhibitor potently downregulated cATF6 and KIT levels (Fig. 5C and Supplementary Fig. S15). In the presence of TG, the anti-tumor effect of each ATF6 inhibitor, with or without ER stress-inducing drugs, was slightly enhanced (Supplementary Fig. S16). In addition, the antitumor effect of PF was evaluated using a panel of cell lines, including a mutant KIT-expressing leukemia cell line (Kasumi), wild type KIT-expressing cell lines (Colo320DM and H128), and KIT-negative cell lines (HeLa, Capan-1, and MDA-MB-231). The antitumor effect of PF was found to be minimal, regardless of the presence of TG (Supplementary Fig. S17).

To verify this, GIST xenografts were generated using GIST430 and GIST430-V654A cells. The antitumor effect of PF with or without bortezomib was then assessed, and imatinib was used as a control drug. As expected, the GIST430 xenograft model was sensitive to imatinib, while the GIST430-V654A model was resistant to imatinib. Treatment with PF (30 mg kg^−1^) potently inhibited tumor growth irrespective of imatinib sensitivity. In contrast, treatment with bortezomib alone (1 mg/kg) resulted in a slight suppression of tumor growth, irrespective of imatinib sensitivity. These findings indicate that ER stress induction alone is not sufficient to effectively suppress GIST growth. When combined with bortezomib, the antitumor efficacy was synergistically enhanced, especially in the imatinib-resistant GIST430-V654A model (Fig. 5D). IHC was performed using mouse tissues to measure the protein levels of KIT, Ki67, and cleaved caspase-3. KIT and Ki67 levels gradually decreased in the following order: bortezomib, imatinib, PF, and PF with bortezomib, whereas cleaved caspase-3 showed the opposite (Fig. 5E, F, and Supplementary Fig. S18).

### Nuclear expression of ATF6 is frequently detected in GIST tissues

To demonstrate the clinical significance of ATF6 in GISTs, ATF6 expression in 42 patient tissues was analyzed. IHC analysis revealed that 33% of the patients (14/42, H-score ≤10) showed negative nuclear ATF6 expression, while 67% of the patients (28/42, H-score >10) showed positive nuclear ATF6 expression (Fig. 5G, Supplementary Table S1). Survival analysis indicated that patients with nuclear ATF6 expression had significantly shorter relapse-free survival (*P* = 0.033) and a tendency for shorter overall survival (*P* = 0.339) (Fig. 5H). Moreover, nuclear ATF6 expression showed significant correlation with recurrence or metastasis (*P* = 0.026; Supplementary Table S1). No significant association between KIT mutation and ATF6 expression was observed (data not shown). The KIT mutation status and details of imatinib treatment for the 42 patients are described in Supplementary Table S2.

## Discussion

Gain-of-function mutations in *KIT* are found even in the smallest GISTs and are therefore considered as an early oncogenic milestone of GIST tumorigenesis [20]. Xiang et al. were the first to report that pathogenic KIT signaling occurs from the Golgi. They demonstrated that hKIT^D816V^ becomes trapped in the endoplasmic reticulum (ER) in murine cells but localizes to the Golgi when expressed in human A375 cells. This difference was attributed to species-specific posttranslational modifications [21]. Subsequently, our group and others reported that both imatinib-sensitive and imatinib-resistant MT-KIT accumulate on the Golgi during the early secretory pathway. Furthermore, they found that MT-KIT becomes fully auto-phosphorylated exclusively on the Golgi and only in a complex-glycosylated form. Additionally, Obata et al. reported that the inhibitor of protein trafficking from the ER to the Golgi, 2-methylcoprophilinamide, suppresses KIT autophosphorylation, underscoring the importance of Golgi localization of MT-KIT in GIST tumorigenesis [22].

The previous findings mentioned above raise more questions, such as how MT-KIT fully folds into a functional protein without interruption of ERQC and how it activates downstream signaling in the Golgi complex. Several studies on GISTs have assumed that MT-KIT localized in the PM delivers downstream signaling [23]. However, mutations in membranous proteins often result in unexpected changes in their localization and function. For instance, a cystic fibrosis transmembrane conductance regulator with a phenylalanine deletion at position 508 is targeted by PMQC and is therefore undetectable on the cell surface [11]. Similarly, our findings showed that only a small portion of MT-KIT reached the PM and was rapidly degraded through PMQC. Moreover, inhibition of trans-Golgi-to-PM trafficking did not affect MT-KIT downstream signaling in GIST. These results suggest that membranous MT-KIT shows a loss-of-function phenotype rather than a gain-of-function phenotype, and therefore, membranous MT-KIT is hardly involved in GIST tumorigenesis. These findings may explain why some therapeutic trials using KIT antibody targeting membranous KIT failed to show antitumor effects in GIST xenograft models [24, 25].

The Golgi complex is a specialized organelle for protein processing and trafficking [26]. Currently, little is known about the function of the Golgi complex, specifically as a platform for initiating receptor tyrosine kinase (RTK) signaling. It was demonstrated that MT-KIT directly recruits P85 and GRB2 to the Golgi complex, and its loss in the Golgi complex drastically perturbs downstream signaling. Herein, we propose a Golgi-based signaling model for MT-KIT. Since the Golgi complex is not central to protein quality control, this unique form of signaling that hides RTK in the Golgi complex would be advantageous for cancer cells, as there is always a risk that mutant RTKs in the ER or PM are degraded through ERQC and PMQC.

Mutations in *KIT* are most frequently detected in exon 11 (juxtamembrane domain, 70%), followed by exon 9 (extracellular domain, 10%) and less frequently in exon 13 (adenosine triphosphate (ATP)-binding pocket, 1%), or in exon 17 (activation loop, 1%) [27, 28]. Since GIST430, GIST882, GIST48, and GIST430-V654A cells commonly displayed Golgi-localization of MT-KIT regardless of mutation type, we suspected that GIST might have acquired a complementary mechanism for Golgi-retention of MT-KIT. BLZF1, a Golgi resident protein [29], was found to be important in tethering MT-KIT in the Golgi complex and thereby for GIST survival. Considering its selective and high expression in GIST, BLZF1 should be further characterized as a druggable target for GIST treatment.

To rationalize the localization of MT-KIT in the Golgi complex, ERQC bypass of MT-KIT should be preceded. As a large solid tumor, GIST undergoes chronic ER stress conditions, such as insufficient nutrients and hypoxia, that eventually induce ERQC and cell death [30–32]. Cancer cells alleviate ER stress by initiating the UPR system that usually leads to mutant protein degradation by ERQC. However, this mechanism might be disadvantageous for cancer cells because of the loss of driver oncoproteins, such as MT-KIT [33, 34]. Therefore, promoting folding rather than degrading mutant proteins may be favorable for long-term cancer progression. Results of this study show evidence that GIST has developed an ATF6-dependent UPR activation mechanism to ameliorate ER stress. Sustained activation of ATF6 was detected in all GIST cell lines and frequently in the patients’ tissues. This unique ER stress-protective environment allows GISTs to resist ER stress to some extent. Pharmacological inhibition of ATF6 significantly decreased chaperone expression, including HSP90, and resensitized GIST cells, even under mild ER stress. The ATF6-HSP90 axis is especially beneficial for GIST tumorigenesis in a manner of MT-KIT folding and ERQC bypass of MT-KIT. This finding is consistent with previous research showing that 17AAG and TAS-116, HSP90 inhibitors, effectively suppress GIST growth [21]. HSP90 inhibitors have a different mechanism of action from kinase activity inhibition, such as that seen with imatinib. Instead, they fundamentally downregulate mutant KIT expression. This mechanism sets them apart from imatinib, as it makes these drugs irrelevant to the generation of secondary mutations that can weaken the antitumor effect. By targeting mutant KIT expression itself, HSP90 inhibitors offer a promising approach for GIST treatment that may overcome imatinib resistance mechanisms associated with secondary mutations. Moreover, previous studies have suggested that sustained activation of ATF6 is a prosurvival mechanism in various cancer types such as melanoma, glioblastoma, and hepatocellular carcinoma [35–37]. Based on these findings we propose that HSP90 inhibitors, either alone or in combination with ATF6 inhibitors, hold great promise as alternative treatments for GIST.

In vitro and in vivo experiments clearly demonstrated that ATF6 inhibition is a promising strategy for GIST treatment, and combined treatment with ER stress-inducing drugs further enhances the antitumor efficacy. This treatment strategy showed invariable effects even on imatinib-resistant GIST, which is a major challenge in clinical practice. Although there are no FDA-approved regimens for targeting ATF6 in cancers, three reported ATF6 inhibitors with different modes of action have shown great efficacy [35, 38, 39]. Notably, melatonin, a relatively safe dietary supplement, effectively suppressed GIST growth at a generally safe concentration (1 mM) and showed synergistic effects with ER stress-inducing drugs. Although melatonin use for GIST treatment requires intensive in vivo and clinical validation, this strategy could be practical since no severe side effects were reported for melatonin. Considering that sustained activation of ATF6 is exclusively observed in GIST and that the ATF6-HSP90 axis plays a crucial role in MT-KIT stabilization, we propose that ATF6 is a promising multi-effect target for GIST treatment, at least for imatinib-resistant patients with no other therapeutic options.

**Fig. 6:**
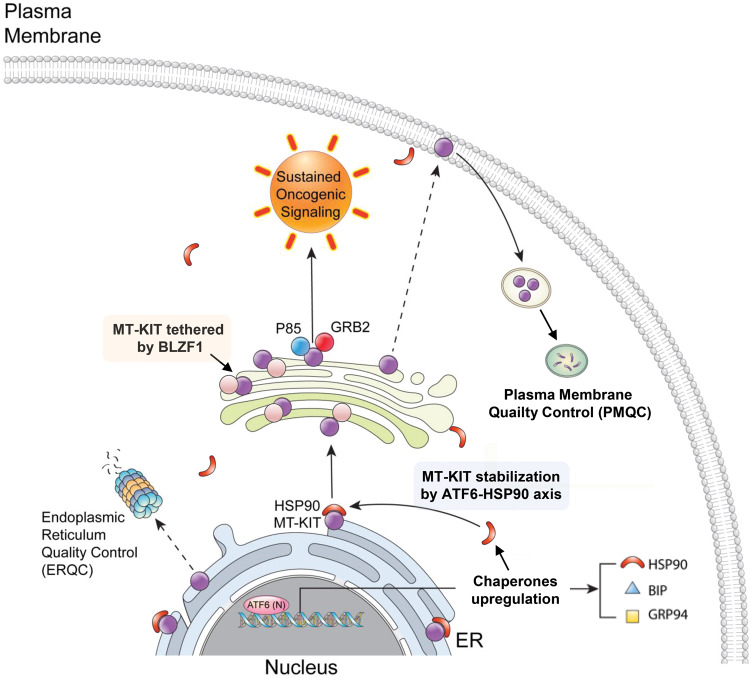
Schematic representation of novel GIST tumorigenesis mechanisms via the Golgi-localized MT-KIT-mediated oncogenic signaling and constant activation of pro-survival ATF6 dependent UPR signaling.

In conclusion, this study provides evidence that GIST has developed highly coordinated survival strategies of hiding MT-KIT in the quality control-free Golgi complex, and developing an intrinsic ER stress resistance mechanism (summarized in Fig. 6). Our findings provide insights into future GIST treatment strategies. Therapeutic antibodies targeting membranous MT-KIT might be challenging. BLZF1 and ATF6 pathway could be promising drug targets for GIST treatment.

In-depth mechanistic and clinical studies are crucial to enhance our understanding of whether targeting BLZF1 and ATF6 could overcome the limitations of conventional therapy in actual patients with GISTs. Additionally, investigating the relationship between BLZF1 and ATF6 would provide valuable insights into their cooperative roles in GIST pathogenesis. It would be beneficial to utilize genomic and transcriptomic analysis data from patients with GIST to further advance our understanding. Furthermore, leveraging the differential protein trafficking regulation between murine and human cells can be a valuable strategy for future studies in this field [40]. By incorporating these approaches, we can gain valuable insights that may lead to the development of improved therapeutic strategies for GIST patients.

## Materials and methods

### Cell culture and treatments

GIST430 (exon 11 in-frame deletion) and GIST430-V654A (exon 11 in-frame deletion and V654A) cells were cultured in Iscove’s modified Dulbecco’s medium (IMDM) supplemented with 15% fetal calf serum (Life Technologies, Carlsbad, CA, USA). GIST48 (V560D) cells were cultured in IMDM supplemented with 15% fetal bovine serum (FBS; Life Technologies). GIST882 (homozygous exon 13 K642E point mutation) cells were cultured in RPMI1640 with 15% FBS. GIST cell lines were established by Prof. Jonathan Fletcher (Boston, MA, USA). Colon cancer cell lines (DLD-1, LS174T, Colo320DM) and small-cell lung cancer cell lines (H69, H128, H209) were cultured in RPMI1640 medium supplemented with 10% FBS. The leukemia cell lines K562 and Kasumi-1 were cultured in RPMI1640 medium supplemented with 10% FBS. Another leukemia cell line, HMC-1, was cultured in IMDM medium supplemented with 15% FBS. A cervical cancer cell line (HeLa), pancreatic cancer cell line (Capan-1), and breast cancer cell line (MDA-MB-231) were used as KIT-negative cell lines. HeLa cells were cultured in Minimum Essential Medium supplemented with 10% FBS. Capan-1 and MDA-MB-231 cells were cultured in RPMI1640 medium supplemented with 10% FBS. Cell lines were authenticated by *KIT* sequencing and microscopic examinations. RT-qPCR and western blot was performed to monitor morphology, growth patterns, mutation status, and KIT expression. Cell lines were screened bi-monthly to monitor for mycoplasma infections.

For treatment reagents, GIST cells were treated with thapsigargin (TG; Sigma, St. Louis, MO, USA), bortezomib (Sigma), or 17AAG (Sigma) to mimic endoplasmic reticulum (ER) stress; Brefeldin A (BFA; Sigma) to inhibit ER-to-Golgi trafficking; Bafilomycin A1 (BAF; Sigma) to inhibit lysosomal degradation; cycloheximide CHX) to block translation; 30N12 (Chembridge, San Diego, CA, USA) to inhibit trans-Golgi-to-PM trafficking. PF429242 (Tocris, Ellisville, MO, USA), Ceapin-A7 (Sigma), and melatonin (Sigma) were used to inhibit ATF6 activation. Imatinib mesylate (Sigma) was used as the control drug for GIST treatment.

### Construction of expression vectors

To construct GRB2 and p85 expression vectors with a hemagglutinin (HA) tag, GRB2 and p85 coding regions were amplified by PCR using cDNA from GIST430 cells, then cloned into a pCMV vector with an HA tag. The primers used to construct GRB2 and P85 expression vectors are listed in Supplementary Table S3.

### Western blot and immunoprecipitation

Whole cell lysates were prepared using passive lysis buffer (Promega, Madison, WI, USA) with a protease inhibitor cocktail (Roche, Basel, Switzerland). The proteins were electrophoresed and transferred onto a membrane. The membranes were incubated overnight at 4 °C with primary antibodies against GAPDH (Trevigen, Gaithersburg, MD, USA), KIT (Dako or Cell Signaling Technology, Beverly, MA, USA), HA (Cell Signaling Technology), phospho-KIT (Cell Signaling Technology), AKT (Cell Signaling Technology), phospho-AKT (Cell Signaling Technology), ERK (Santa Cruz Biotechnology, Santa Cruz, CA, USA), phospho-ERK (Santa Cruz Biotechnology), P85 (Cell Signaling Technology), GRB2 (Cell Signaling Technology), GBF1 (BD Biosciences; NJ, USA), GM130 (BD Biosciences), Golgin97 (MyBioSource, San Diego, CA, USA), TGN38 (Bio-Rad Laboratories, Hercules, CA, USA), GRASP55 (Abcam), GRASP65 (Invitrogen, Carlsbad, CA, USA), BLZF1 (Novus Biologicals, Littleton, CO, USA), STX3 (Abcam), STX6 (Cell Signaling Technology), GOLPH3 (LifeSpan Biosciences, Seattle, WA, USA), ATF6 (Abcam), IRE1α (Cell Signaling Technology), phospho-IRE1α (Novus Biologicals), PERK (Cell Signaling Technology), phospho-PERK (Cell Signaling Technology), CHOP (Cell Signaling Technology), BIP (Cell Signaling Technology), GRP94 (Cell Signaling Technology), HSP70 (Abcam) and HSP90 (Cell Signaling Technology). The membranes were washed and incubated with the appropriate secondary antibodies for 1 h at room temperature. Western blot images were analyzed using an LAS 4000 mini camera (Fujifilm, Tokyo, Japan). For immunoprecipitation assays, immune complexes of MT-KIT were collected by mixing 500 µg of GIST cell lysate with a KIT antibody (Cell Signaling Technology) and protein A/G PLUS-Agarose (Santa Cruz Biotechnology), then gently rocking on an orbital shaker at 4 °C overnight. Immune complexes bound to agarose beads were washed and boiled with 3× NuPAGE LDS Sample Buffer (Thermo Fisher Scientific) to elute the complexes. The relative density of each band was quantified using ImageJ software (Ver 1.53 K; National Institutes of Health, MD, USA).

### Tissue sample and immunohistochemistry

Formalin-fixed paraffin-embedded (FFPE) GIST tissue samples from 42 patients were used. All specimens were obtained via surgical resection, and some of the clinicopathological findings of the 42 GIST samples have been previously reported [4]. The specimens were obtained from the archives of the Department of Pathology, Yonsei University, Seoul, Korea and the Liver Cancer Specimen Bank of the National Research Resource Bank Program of the Korea Science and Engineering Foundation of the Ministry of Science and Technology. Authorization for the use of these tissues for research purposes was obtained from the Institutional Review Board of Yonsei University, College of Medicine (IRB 4-2015-0227). For IHC analysis, 4 µm-thick sections were obtained from FFPE GIST tissue specimens obtained from 42 patients. IHC was performed using the Ventana Discovery XT autoimmunostainer (Ventana, Tucson, AZ, USA) with antibodies against KIT (1:200, Dako) and ATF6 (1:200, Abcam) according to the manufacturer’s standard protocol. IHC results were evaluated using an H-score method. The H-score method, which assigns an IHC H-score to each case on a continuous scale of 0–300 based on the percentage of cells at different staining intensities, was used for the interpretation of KIT and ATF6 results. The staining intensity was scored as follows: 0 = no staining; 1+ = weak staining visible at high magnification; 2+ = intermediate staining; 3+ = strong staining. The percentage of cells at different staining intensities was determined by visual assessment with scores calculated using the following formula: 1(% of 1+ cells) +2 (% of 2+ cells) +3 (% of 3+ cells). Results were then classified as low (H-score ≤10) or high (H-score >10) protein level.

### Biotinylation assay

DLD-1, Colo320DM, GIST430, and GIST882 cells were biotin-labeled for 30 min, washed extensively, and harvested for further analysis. To analyze membranous MT-KIT stability, GIST cells were harvested at predefined incubation times. Subsequently, the labeled proteins were isolated and eluted using the Pierce Cell Surface Protein Isolation Kit (Thermo Fisher Scientific, Waltham, MA, USA) according to the manufacturer’s instructions. Because of the low expression of membranous MT-KIT in GIST cells, biotinylation assays for GIST cells were performed with twice the amount of lysate compared to those used for DLD-1 and Colo320DM cells.

### Immunofluorescence

Cells grown on slides were rinsed with phosphate-buffered saline (PBS), fixed with 4% paraformaldehyde or 100% methanol for 15 min, and permeabilized in 0.2% Triton X-100 in PBS. The slides were incubated overnight at 4 °C with primary antibodies against KIT (Cell Signaling Technology), calnexin (Cell Signaling Technology), GM130 (Cell Signaling Technology), mannosidase II (Abcam), Golgin-97 (Cell Signaling Technology), HA (Cell Signaling Technology), BLZF1 (Novus Biologicals), LAMP1 (Cell Signaling Technology), and ATF6 (Abcam). The slides were then incubated for 1 h with the appropriate fluorescence-labeled secondary antibody (Life Technologies). All images were captured using an LSM700 confocal microscope (Carl Zeiss, Oberkochen, Germany). Immunofluorescence intensity and colocalization among proteins were quantified using ImageJ software (NIH).

### Internalization and decay analysis of MT-KIT

GIST cells grown on slides were washed three times with ice-cold serum-free medium and incubated with KIT antibody conjugated with a fluorescent dye (Alexa Fluor 488, Cell Signaling Technology) in ice-cold serum-free medium for 30 min. The cells were washed with cold serum-free medium to remove excess antibodies and incubated at 37 °C for various timepoints. Cells were fixed in 4% paraformaldehyde in PBS and processed for immunofluorescence analysis. Colocalization of MT-KIT with EEA1 (Abcam), LAMP1 (Cell Signaling Technology), and GM130 (Cell Signaling Technology) was examined.

### Cell viability assay

Cell viability was analyzed using the 3-(4,5-dimethylthiazol-2-yl)-2,5-diphenyl-2H-tetrazolium bromide (MTT) assay. GIST cells were seeded in 96-well plates at a density of 3 × 10^4^ cells/well and treated with PF429242, Ceapin-A7, or melatonin with or without thapsigargin, bortezomib, 17AAG, or imatinib for 72 h. MTT (5 mg ml^−1^) solution was then added to the plates and incubated at 37 °C for 4 h. After the formazan crystals were dissolved in DMSO, the OD was measured at 570 nm using a microplate reader. This experiment was performed with or without TG. Highest single agent (HSA) synergy scores were calculated using SynergyFinder 2.0 (10.1093/nar/gkaa216).

### shRNA for gene silencing

Short hairpin RNA (shRNA) against BLZF1 (gene ID: 8548) was purchased from the shRNA library of the RNAi Consortium (TRC) provided by the Yonsei Genome Center (Seoul, Korea). GIST cells were transfected with the TRC2-pLKO-puro vector containing shRNA for BLZF1, and the MTT assay was performed 72 h after transfection.

### Mouse xenograft and drug treatment

All animal modeling procedures were approved by the Institutional Animal Care and Use Committee of the Catholic University of Korea (No. 2022-0037-05). Seven-week-old male BALB/c-nude mice were purchased from Orient Bio (Gyeonggi, South Korea). A total of 1 × 10^7^ GIST430 or GIST430-V654A cells were mixed with an equal volume of Matrigel (BD Biosciences), and each mouse was subcutaneously injected with 0.1 ml of the cell-Matrigel mixture into the flank. Tumor volume was measured using a digital Vernier caliper (Mitutoyo; Kanagawa, Japan) three times a week after implantation and was calculated as 1/2 × length × width^2^ [41]. When the tumor volume had reached 200–300 mm^3^ about 3 weeks, the mice were randomized into five groups (*n* = 4–6/group) according to cell line and administered an intraperitoneal injection of 2% DMSO in PBS (vehicle), 50 mg kg^−1^ imatinib, 30 mg kg^−1^ PF429242, 1 mg kg^−1^ bortezomib, 30 mg kg^−1^ PF429242, and 1 mg kg^−1^ bortezomib. Eight injections were performed at three-day intervals, for a total of eight injections. Body weight and tumor volume were measured three times a week after drug treatment. At the end of the experiments, the animals were sacrificed, and the tumors were isolated, weighed, and stored at −80 °C until further analyses.

### Immunohistochemistry with mouse samples

Tumors were paraffin-embedded and 4 μm sections were used for immunohistochemistry. The sections were deparaffinized and treated with BLOXALL endogenous blocking solution (Vector Laboratories, CA, USA) to inhibit endogenous peroxidase activity. The epitope retrieval was conducted with Retrieve-All Antigen 1 (pH 8), followed by blocking with PBS supplemented with 2% normal horse serum and 3% BSA. Sections were incubated with individual primary antibodies at 4 °C overnight and with the appropriate secondary antibody at room temperature for 1 h. Peroxidase activity was revealed using 3,3-diaminobenzidine (Vector Laboratories), and sections were counterstained with hematoxylin (Vector Laboratories). After dehydrogenation with alcohol and xylene, sections were viewed and photographed with an Olympus BX51 microscope (Olympus, Tokyo, Japan) and Mosaic2.1 software. The following antibodies were used: Ki-67 (1:100, Abcam, Cambridge, UK), cleaved caspase-3 (1:100, Cell Signaling Technology, MA, USA), and KIT (1:200, Abcam). Ki-67, cleaved caspase-3, and KIT. Positive staining in xenografted tumors were quantified using ImageJ software (NIH). Three HPF (high-power fields, at ×400 magnification) were used for each tumor [42].

### Statistical analysis

All data were analyzed using GraphPad Prism 7 (GraphPad Software Inc., La Jolla, CA, USA) and expressed as mean ± SEM. A comparison of the means among groups was performed using Mann-Whitney U test for body weight, tumor weight, and tumor volume data, or one-way analysis of variance (ANOVA) followed by Dunnett’s post-hoc test for IHC data. Fisher’s exact test, Student’s *t* test, and Kaplan–Meier survival analysis were used to analyze the relationship between nuclear ATF6 expression and clinicopathological parameters (SPSS software, SPSS, Inc., Chicago, IL, USA). Statistical significance was set at *P* < 0.05.

### Supplementary information

Supplementary Figures and Tables

Original Data File

Reproducibility checklist

## Data Availability

All data are present in the manuscript and the Supplementary Materials. Additional data related to this paper may be requested from the corresponding author. Original western blot was uploaded as Supplementary Material.
