# Supplementary material for: Identification of novel pathogenic roles of BLZF1/ATF6 in tumorigenesis of gastrointestinal stromal tumor showing Golgi-localized mutant KIT
Source: Cell Death Differ. 2023 Sep 13;30(10):2309–21. doi: 10.1038/s41418-023-01220-2 (PMC10589262; doi:10.1038/s41418-023-01220-2)
Supplement: Supplementary file 1 — Supplementary Figures and Tables [file 41418_2023_1220_MOESM1_ESM.pdf]

Supplementary Figures

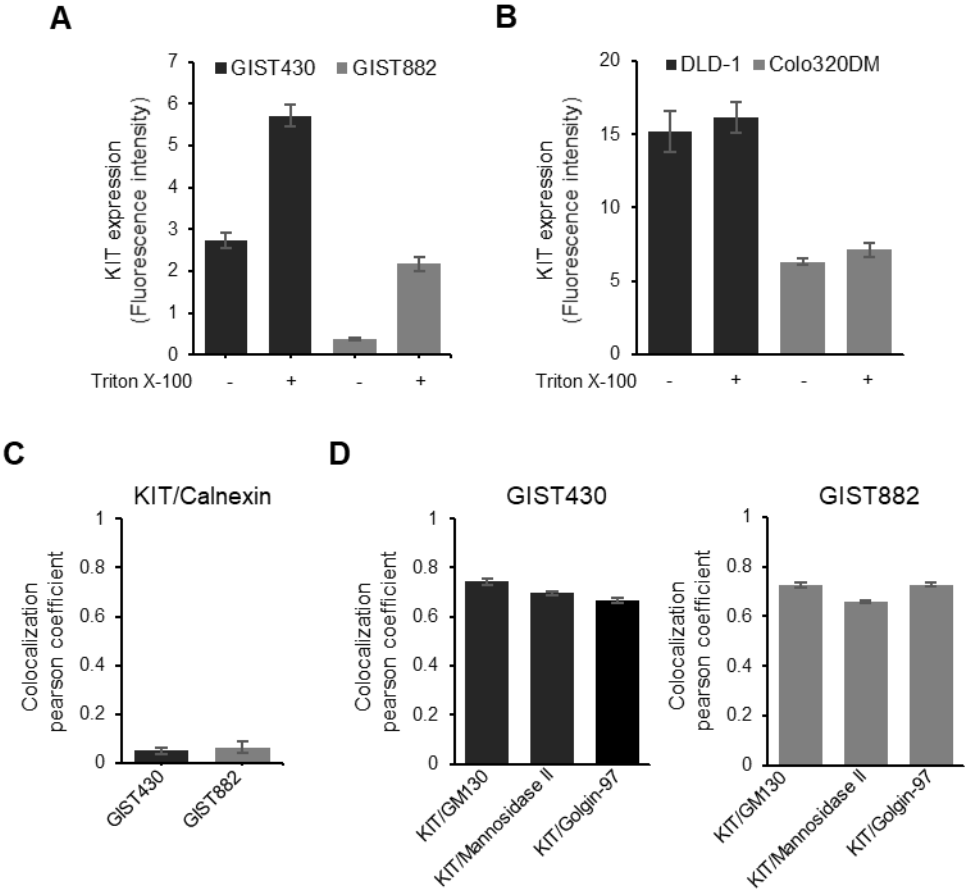

Supplementary Figure S1. Quantification of immunofluorescent intensity of KIT and ER/Golgi markers reveals the localization of MT-KIT to the Golgi complex. (A and B) The immunofluorescent intensities of MT-KIT and WT-KIT, as shown in Figure 1A and 1B, were quantified. **(C and D)** The extent of colocalization of MT-KIT with an ER marker (Calnexin, as shown in Figure 1C) and Golgi markers (GM130, Mannosidase II, Golgin-97, as shown in Figure 1D) was quantified. Pearson correlation coefficient (summarized signal) values >0.5 indicate a high probability that pixels from both channels overlay. Immunofluorescence intensity and colocalization analysis were quantified using ImageJ software.

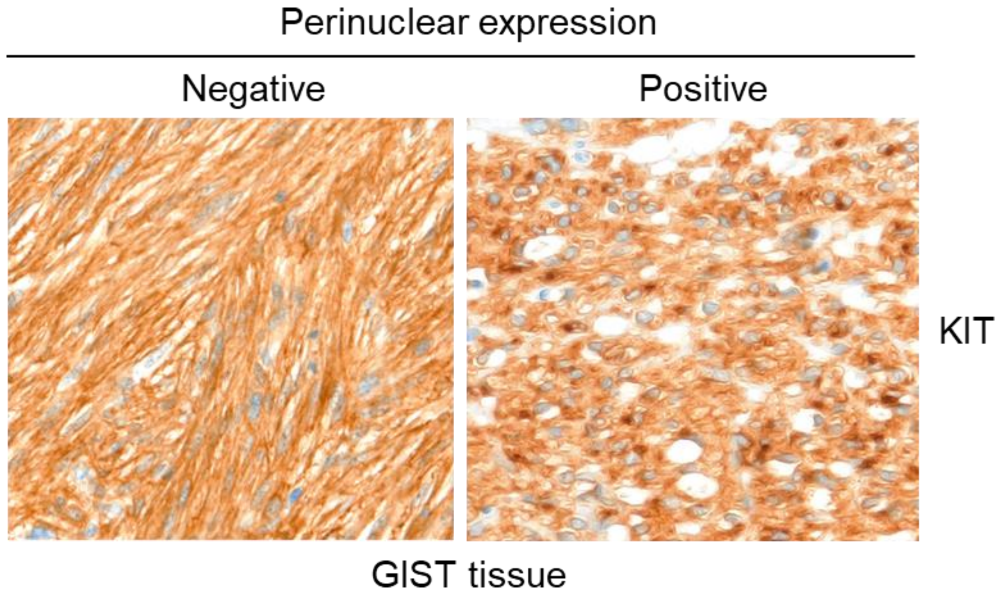

Supplementary Figure S2. GIST patients frequently show perinuclear mutant KIT (MT-KIT) expression. Immunohistochemistry analysis of tissues from patients with GIST (n = 42) was performed to evaluate the perinuclear expression of MT-KIT. The perinuclear expression status was classified into negative and positive categories.

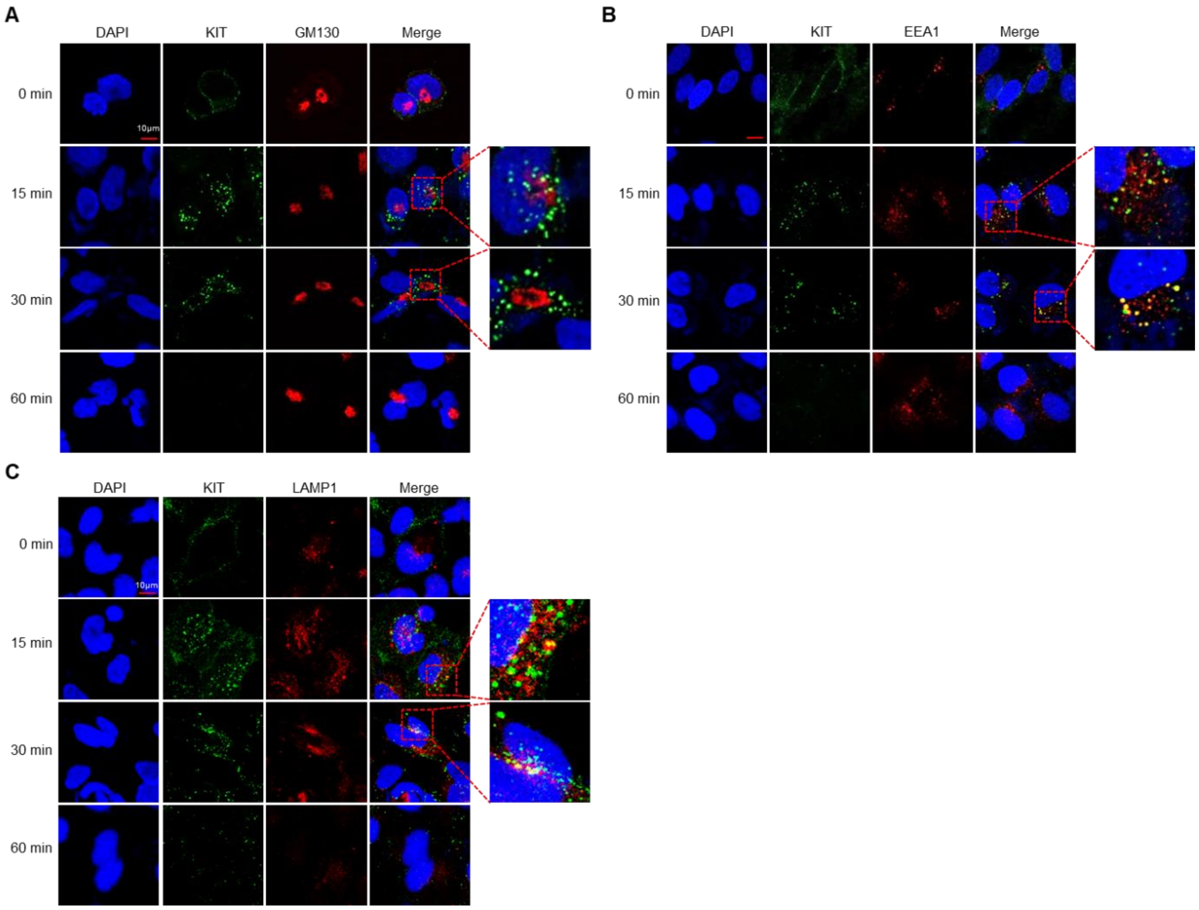

Supplementary Figure S3. Membranous MT-KIT is internalized and degraded without retrieval to the Golgi complex. (A-C) Time-course confocal microscopic analysis of KIT, GM130 (a cis-Golgi marker), EEA1 (an early endosome marker), and LAMP1 (a lysosome marker) in GIST882 cells. GIST cells were labeled with a KIT antibody conjugated with a fluorescent dye, incubated for the indicated times, and analyzed for immunofluorescence.

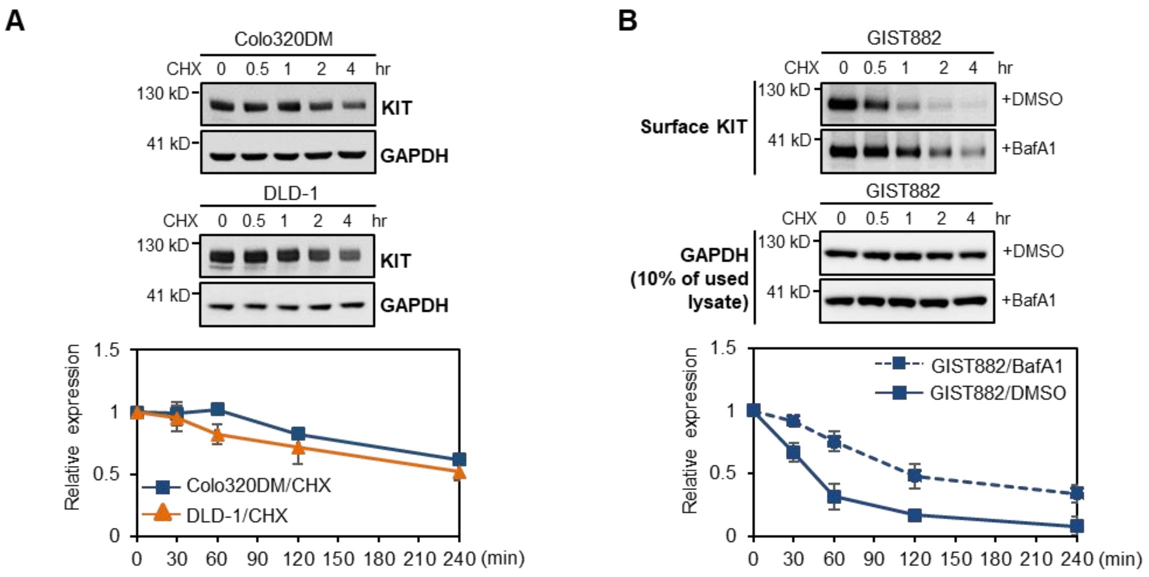

Supplementary Figure S4. Membranous MT-KIT is rapidly degraded by the plasma-membrane quality-control system. (A) The stability of WT-KIT over time was measured by western blotting after treatment of DLD-1 and Colo320DM cells with CHX. **(B)** The stability of MT-KIT over time was measured by biotin-labeling surface proteins, streptavidin pull-down, and western using GIST882 cells treated with or without a lysosomal inhibitor, Bafilomycin A1 (BafA1). Error bars in A and B represent the SD of the mean of three independent experiments.

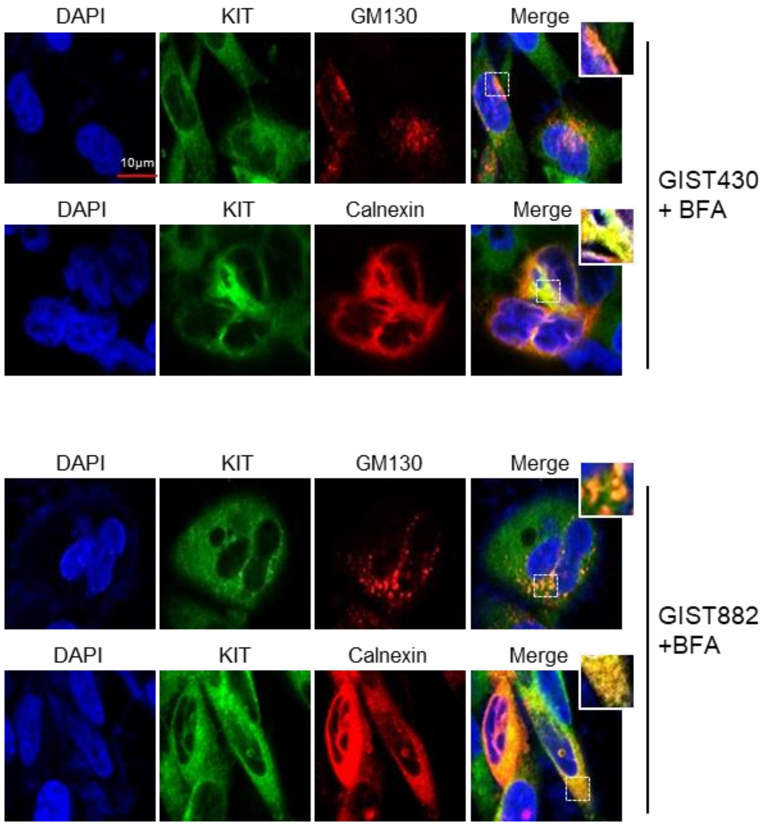

Supplementary Figure S5. Brefeldin A, which blocks ER to Golgi trafficking, leads to ER retention of MT-KIT and disrupts the Golgi structure. Confocal microscopic analysis of GIST cells treated with Brefeldin A (BFA). GM130, KIT, and Calnexin (an ER marker) were examined.

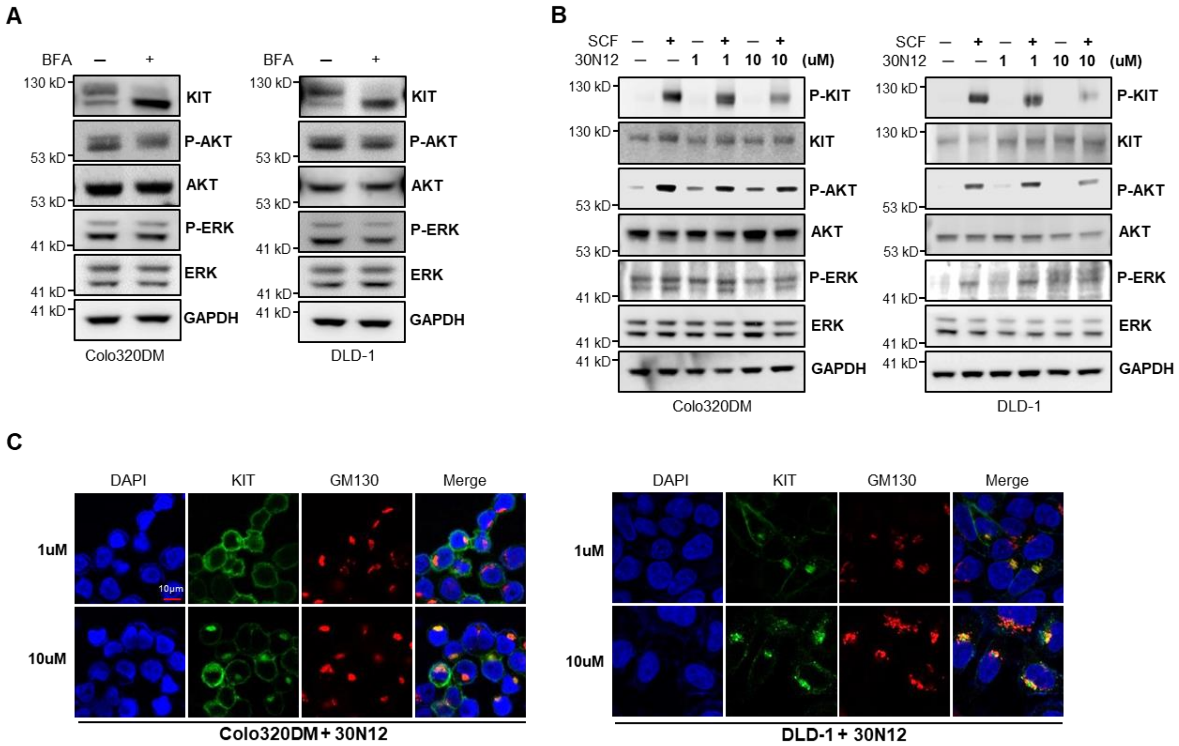

Supplementary Figure S6. 30N12, an inhibitor of trans-Golgi-to-plasma-membrane trafficking, causes retention of WT-KIT in the Golgi complex and perturbs SCF-activated KIT downstream signaling. (A) Western blot analysis of KIT downstream signaling was performed after treating DLD-1 and Colo320DM cells with 5 μg/mL BFA for 4 hours. **(B)** Cells were treated with 1 μM or 10 μM 30N12 for 18 hours. Western blotting was conducted to analyze KIT downstream effector molecules in SCF-activated DLD-1 and Colo320DM cells, both with and without pre-treatment with 1 μM or 10 μM 30N12. **(C)** Confocal microscopy was used to examine KIT and GM130.

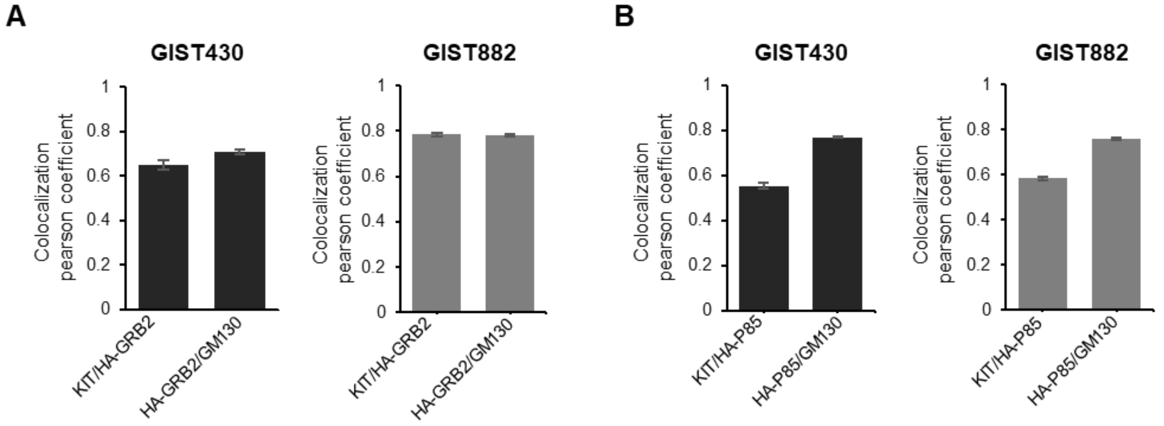

Supplementary Figure S7. GRB2 and P85 evidently colocalize with MT-KIT and GM130 at the Golgi complex. The extent of colocalization of GRB2 and P85 with MT-KIT and GM130 in GIST430 and GIST882 cells was quantified using the images shown in Figure 2D and 2E. Pearson correlation coefficient (summarized signal) values >0.5 indicate a high probability that pixels from both channels overlay. Immunofluorescence intensity and colocalization analysis were quantified using the ImageJ software.

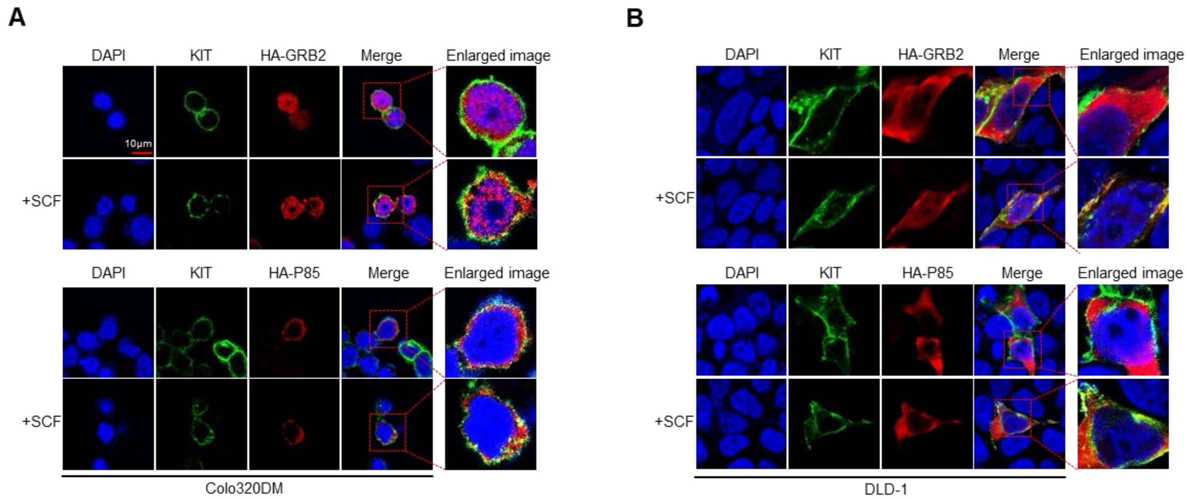

Supplementary Figure S8. WT-KIT activates downstream signaling pathways at the plasma membrane by recruiting P85 and GRB2 to the plasma membrane. (A and B) After SCF treatment, confocal microscopic analysis was performed on DLD-1 and Colo320DM cells transfected with a HA-GRB2 or HA-P85 expression vector. GRB2 is the most upstream molecule of the MAPK/ERK pathway, and P85 is that of the PI3K/AKT pathway.

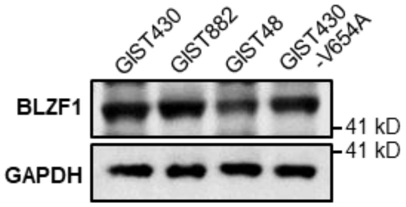

Supplementary Figure S9. GIST cell lines show high BLZF1 expression regardless of mutation status.

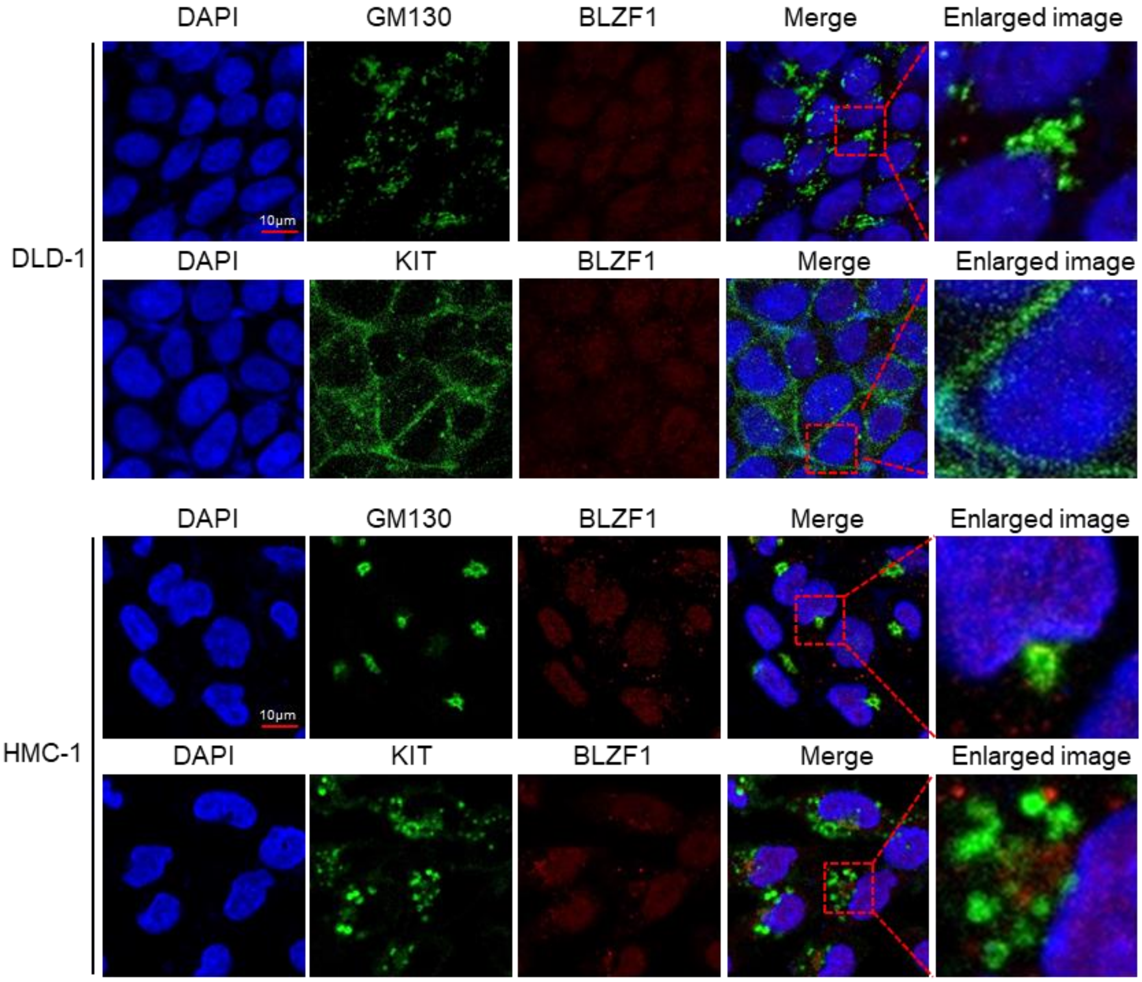

Supplementary Figure S10. Colocalization of KIT with BLZF1 is not observed in CC (DLD-1) and leukemia (HMC-1) cells expressing WT-KIT and MT-KIT, respectively. Confocal microscopic analysis of KIT, GM130, and BLZF1 was performed in DLD-1 and HMC-1 cells.

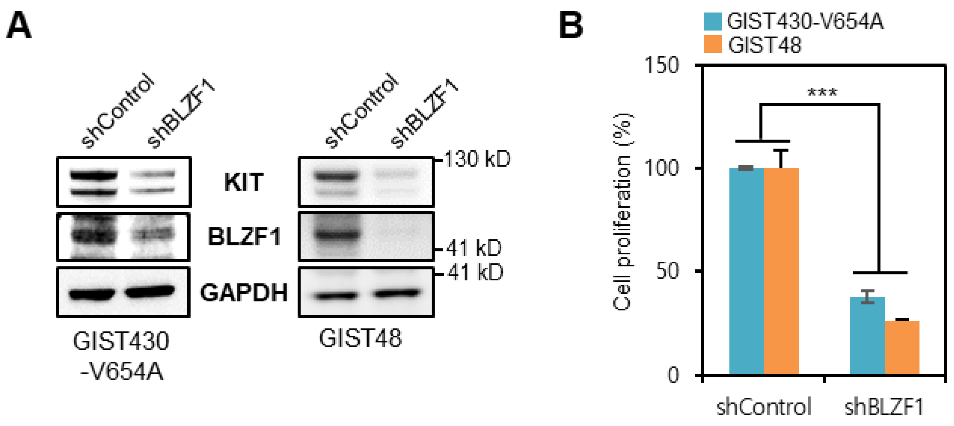

Supplementary Figure S11. BLZF1 knock-down strongly suppresses KIT expression and growth of imatinib-resistant GIST cells. (A) Expression of KIT and BLZF1 were measured by western blotting in imatinib-resistant GIST cell lines (GIST48 and GIST430-V654A) treated with BLZF1 shRNA. **(B)** Proliferation of GIST48 and GIST430-V654A cells was measured by MTT assay after 72 h of treatment with BLZF1 shRNA. Error bars in B represent the SD of the mean of three independent experiments. One-way ANOVA with a post-hoc test was performed to compare multiple means (*$p < 0.05$, **$p < 0.01$, ***$p < 0.001$).

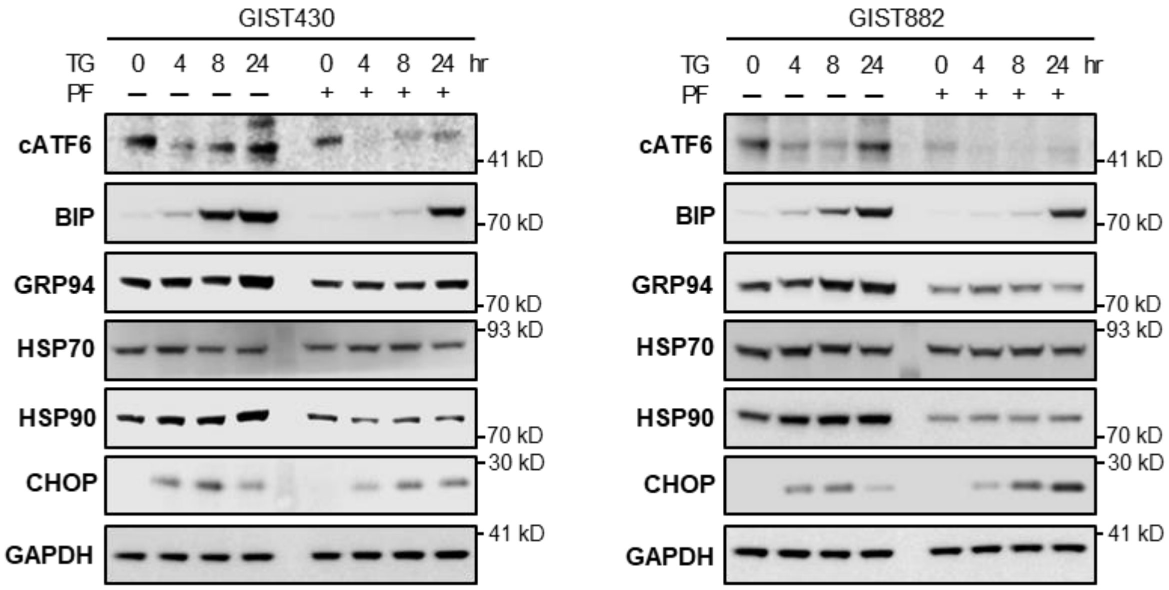

Supplementary Figure S12. ATF6 inhibition delays the expression of chaperones in response to mild ER stress and subsequent ER-stress-mediated cell death. GIST cells were resensitized to mild ER stress (0.1 μM thapsigargin, TG) when the ATF6 pathway was inhibited by PF429242 (PF). The expression of chaperones (BIP, HSP70, HSP90, and GRP94) and a cell-death marker (CHOP) over time was measured by western blotting following 0.1 μM TG treatment with or without PF.

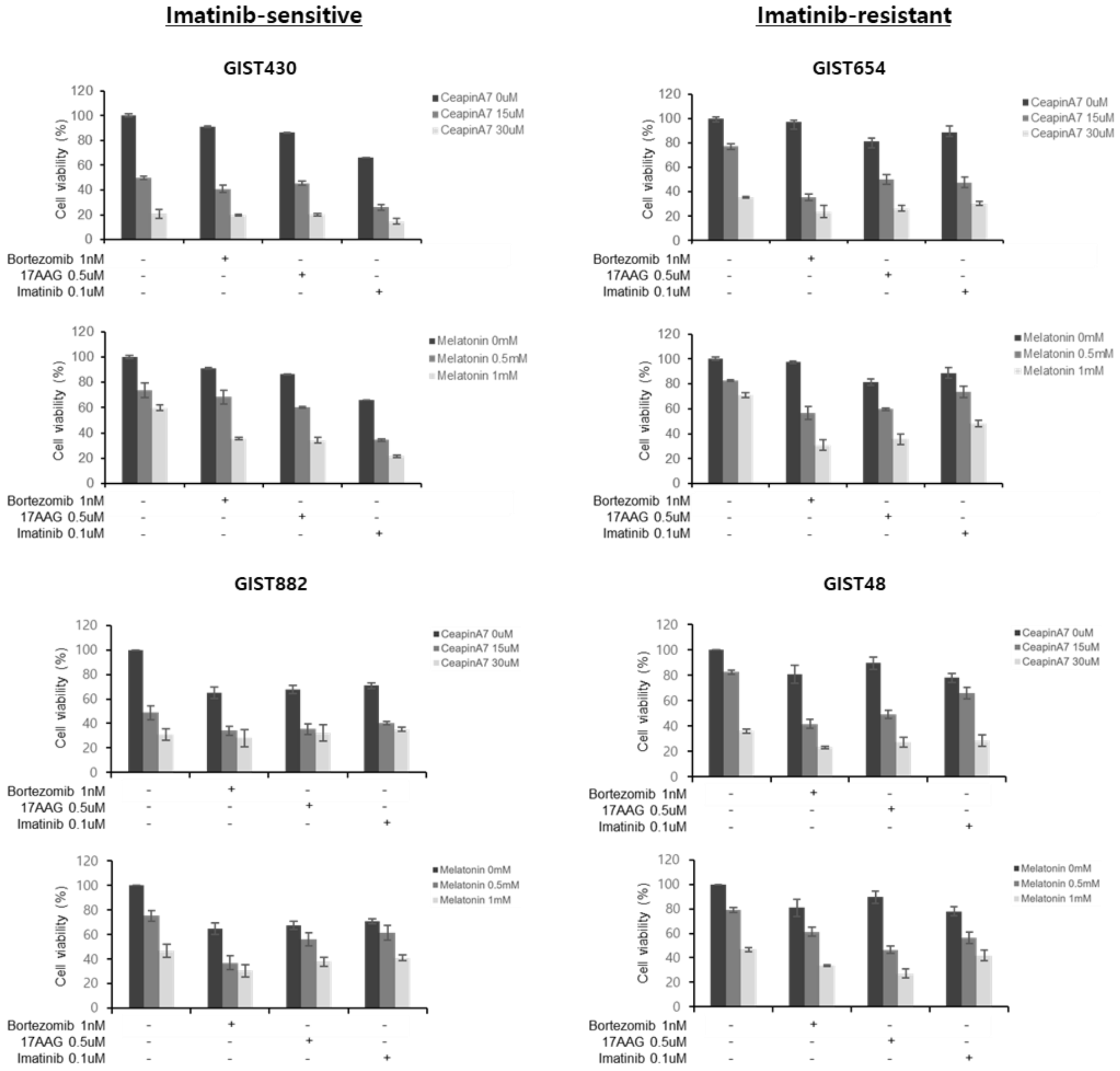

Supplementary Figure S13. ATF6 inhibition efficiently suppresses GIST cell growth, irrespective of imatinib resistance and synergistic anti-tumor effect was observed when combined with ER stress-inducing drugs. Cell viability was measured by MTT assay in imatinib-sensitive GIST430 and GIST882 cells, and imatinib-resistant GIST48 and GIST430-V654A cells. GIST cells were treated with an ATF6 inhibitor alone (15 μM or 30 μM Ceapin-A7 and 0.5 mM or 1 mM melatonin) or with an ATF6 inhibitor and ER stress inducing drugs (0.5 μM 17AAG and 1 nM borteazomib) for 72 h. Imatinib (0.1 μM) was used as a positive control drug and synergistic effect with ATF6 inhibitors was analyzed.

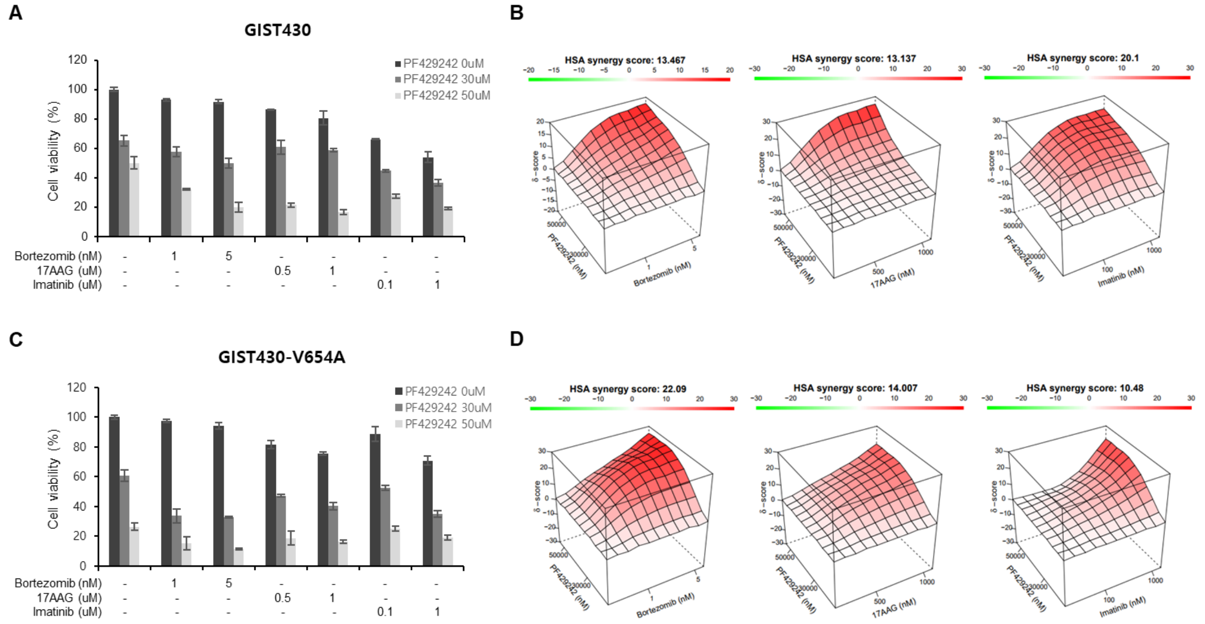

Supplementary Figure S14. PF429242, an ATF6 inhibitor shows significant synergistic effects when combined with bortezomib, 17AAG, and imatinib. Cell viability was assessed using MTT assay in both imatinib-sensitive GIST430 cells and imatinib-resistant GIST430-V654A cells. GIST430 cells **(A and B)** and GIST430-V654A cells **(C and D)** were incubated with two different concentrations of imatinib (0.1 or 1 μM) and ER stress-inducing drugs, 17AAG (0.5 or 1 μM) and bortezomib (1 or 5 nM), in combination with PF429242 (15 μM or 30 μM), for 72 hours. The synergistic effects of each drug with the ATF6 inhibitor were analyzed, and the highest single agent (HSA) synergy scores for each drug with the ATF6 inhibitor were calculated using SynergyFinder 2.0. A HSA synergy score value greater than 10 was considered synergistic, while scores between -10 and +10 were considered additive, and scores below -10 were considered antagonistic.

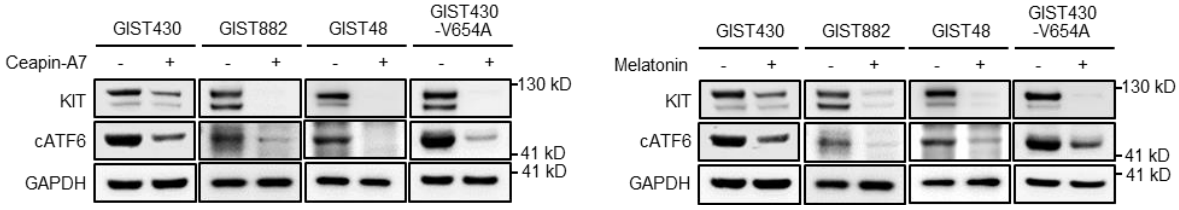

Supplementary Figure S15. Ceapin-A7 and melatonin strongly downregulate ATF6 activation and KIT expression. Western blotting against to ATF6 and KIT was performed in GIST cells treated with 30 μM Ceapin-A7 and 1 mM melatonin for 24 h.

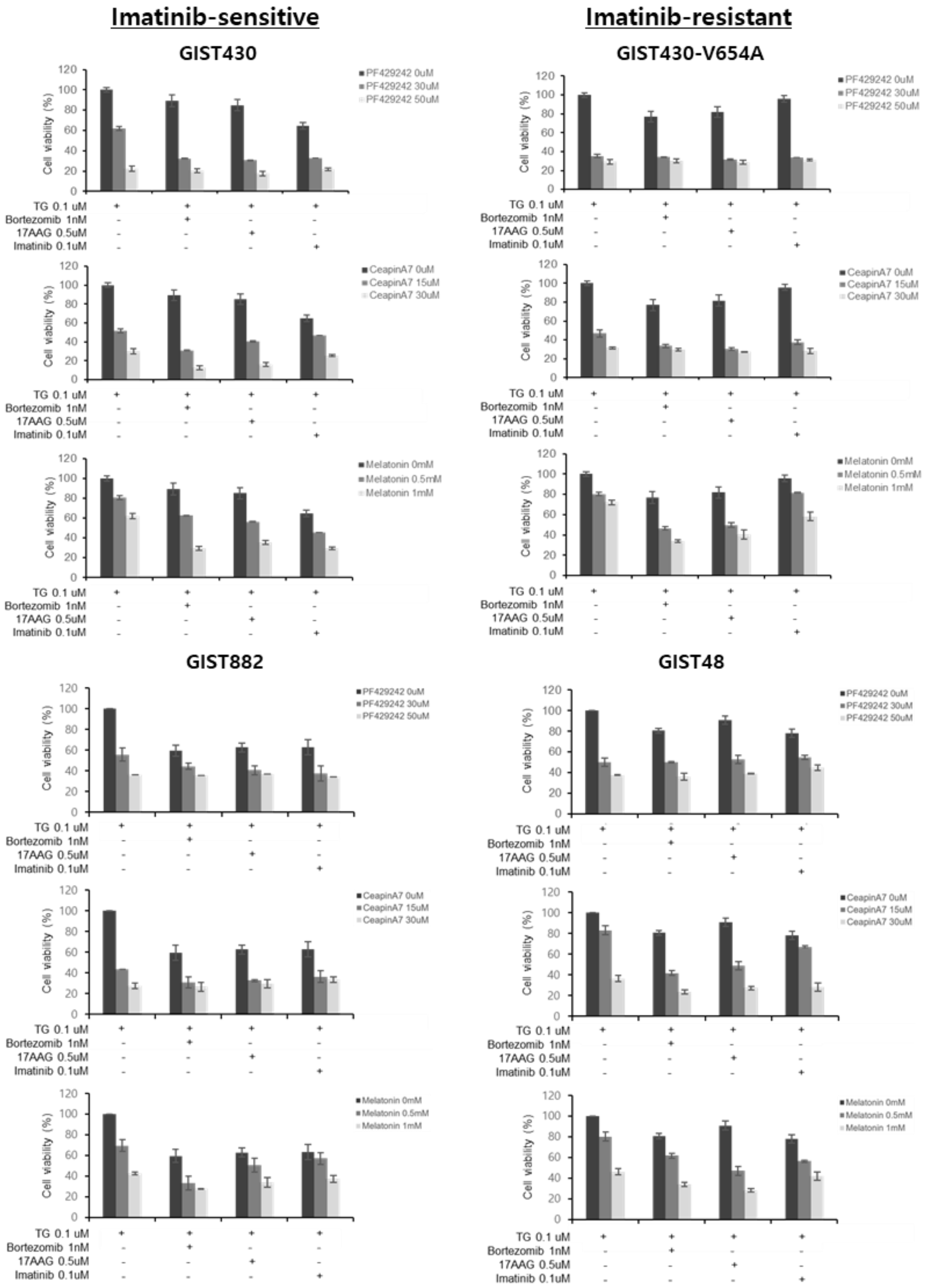

Supplementary Figure S16. Anti-tumor effects of ATF6 inhibition and ATF6 with ER stress-inducing drugs is augmented under thapsigargin treatment. Cell viability was measured by MTT assay in imatinib-sensitive GIST430 and GIST882 cells, and imatinib-resistant GIST48 and GIST430-V654A cells. GIST cells were treated with an ATF6 inhibitor alone (30 μM or 50 μM PF429242, 15 μM or 30 μM Ceapin-A7, and 0.5 mM or 1 mM melatonin)

or an ATF6 inhibitor and ER stress inducing drugs (0.5 μM 17AAG and 1 nM borteazomib) for 72 h. Imatinib (0.1 μM) was used as a positive control drug and synergistic effect with ATF6 inhibitors was analyzed. This experiment was performed under a mild ER stress condition (0.1 μM thapsigargin) to mimic the in vivo tumor microenvironment.

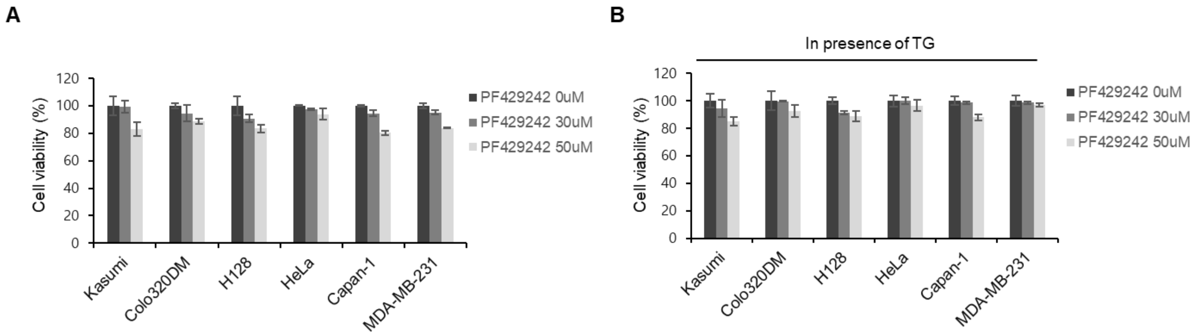

Supplementary Figure S17. ATF6 inhibition barely exhibits cytotoxicity in cell lines other than GIST cells. (A and B) MTT assay was performed to analyze the cell viability in various cell lines: mutant KIT-expressing leukemia cell line (Kasumi), wild-type KIT-expressing cell lines (Colo320DM and H128), and KIT-negative cell lines (HeLa, Capan-1, and MDA-MB-231). The cells were treated with an ATF6 inhibitor (30 μM or 50 μM PF429242) with or without an ER stress-inducing reagent (0.1 μM thapsigargin) for a duration of 72 hours.

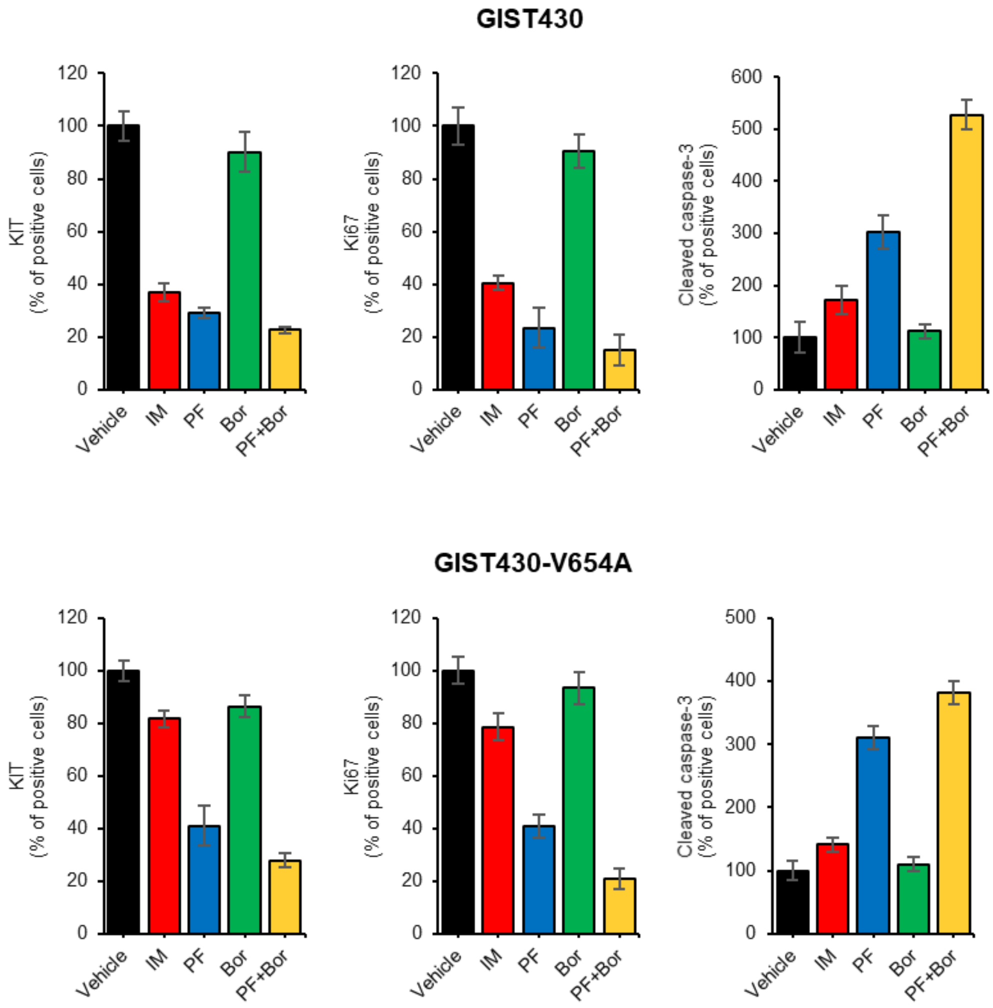

Supplementary Figure S18. Quantification of IHC assay results of KIT, Ki67 and cleaved caspase-3 in tumor tissues from xenograft mouse models. The average number of positive cells was calculated from three fields at 400x magnification.

Supplementary Tables

Supplementary Table S1. Clinicopathological characteristics of the 42 GISTs according to nuclear ATF6 expression status

Category	Variables	Case no. (n=42)	Nuclear ATF6 expression		P value
			positive (%) (n=28)	negative (%) (n=14)	
Age (years)	20-40	6	4 (14.3)	2 (14.2)	0.894
	40-60	16	10 (35.7)	6 (42.9)	
	> 60	20	14 (50)	6 (42.9)	
Gender	Male	16	13 (46.4)	3 (21.4)	0.116
	Female	26	15 (57.7)	11 (78.6)	
Location	Stomach	22	14 (50.0)	8 (57.1)	0.734
	Small intestine	19	13 (46.4)	6 (42.9)	
	Rectum	1	1 (3.6)	0 (0)	
Tumor size (cm)	≤5	18	10 (35.7)	8 (57.1)	0.172
	6-10	19	13 (46.4)	6 (42.9)	
	>10	5	5 (17.9)	0 (0)	
Grade (modified NIH)	Low	16	10 (35.7)	6 (42.9)	0.275
	Intermediate	5	2 (7.1)	3 (21.4)	
	High	21	16 (57.1)	5 (35.7)	
Mitotic count	≤5	19	12 (42.9)	7 (50)	0.451
	6-10	6	3 (10.7)	3 (21.4)	
	>10	17	13 (46.4)	4 (28.6)	
KIT mutation	Absent	6	5 (17.9)	1 (7.1)	0.350
	Present	36	23 (82.1)	13 (92.9)	
KIT perinuclear expression	Negative	14	8 (28.5)	6 (42.9)	0.238
	Positive	28	20 (71.4)	8 (57.1)	
Imatinib response	No treatment	31	19 (67.9)	12 (85.7)	0.234
	Stable disease	6	4 (14.3)	2 (14.3)	
	Progressed disease	5	5 (11.9)	0 (0)	
Recurrence /metastasis	Negative	34	20 (71.4)	14 (100)	0.026
	Positive	8	8 (28.6)	0 (0)	
Survival	Alive	40	26 (92.9)	14 (100)	0.306
	Expired	2	2 (7.1)	0 (0)	

Supplementary Table S2. Mutation status of *KIT* and imatinib treatment in 42 GISTs

Case No.	Age (year)	Gender	Location	*KIT* mutation status	Imatinib response
1	66	M	stomach	K550_V555 del, I ins	NT
2	59	M	small intestine	N564_I571 del, D572Y	NT
3	49	F	stomach	WT	NT
4	68	M	stomach	M552_Y553 del	NT
5	66	M	stomach	W557_K558 del	PD
6	56	M	small intestine	c.1449C>T	NT
7	60	M	stomach	K558_E562 del	PR
8	35	F	small intestine	Q556_V559 del	PD
9	58	F	stomach	L576P	NT
10	54	M	stomach	N557_K558 del	NT
11	46	M	small intestine	A504_Y505 ins	NT
12	65	M	stomach	V560D	NT
13	38	F	small intestine	WT	NT
14	65	F	stomach	WT	PD
15	63	F	stomach	L576P	NT
16	23	F	stomach	T574_K581 del, K ins	NT
17	65	F	small intestine	M552_Y570 del	SD
18	75	M	stomach	Q556_V559 del	SD
19	76	M	small intestine	V559D	SD
20	83	F	stomach	WT	NT
21	27	M	small intestine	W557_V560>C, L800F	NT
22	54	M	stomach	V559D	NT
23	52	F	small intestine	V560D	NT
24	66	F	small intestine	W557_K558del	NT
25	58	F	rectum	WT	PD
26	67	F	small intestine	A502_Y503 ins	NT
27	62	F	small intestine	F509_F511 ins	NT
28	54	F	small intestine	W557_K558del	SD
29	69	F	stomach	K558_I563 del, I ins	NT
30	42	F	stomach	V559D	NT
31	50	M	stomach	W557_K558 del	NT
32	70	F	stomach	V559D	NT
33	73	F	stomach	W557R	SD
34	46	F	small intestine	M552_N572 del	NT
35	21	F	small intestine	W557_K558del, c.1449C>T	NT
36	61	F	stomach	D579 del	SD
37	64	F	stomach	W557_K558 del	NT
38	39	F	stomach	V559D	NT
39	49	F	small intestine	W557_K558 del	NT
40	54	F	small intestine	c.1518T>C	NT
41	59	M	small intestine	V560del	NT
42	69	M	small intestine	WT	NT

F, female; M, male; del, deletion; ins, insertion; WT, wild type; NT, no treatment; SD, stable disease; PD, progressed disease

Supplementary Table S3. Primers used for the construction of expression vectors

Gene	Direction	Sequence
P85	Forward	5'- GTTTTCCCAGTCACGACGTT
	Reverse	5'- TGTGGAATTGTGAGCGGATA
GRB2	Forward	5'- CTTCAGGCTGCTGAGCACT
	Reverse	5'- TGTTCTGCACTCCCTCACAG
