# Supplementary material for: Identification of novel pathogenic roles of BLZF1/ATF6 in tumorigenesis of gastrointestinal stromal tumor showing Golgi-localized mutant KIT
Source: Cell Death Differ. 2023 Sep 13;30(10):2309–21. doi: 10.1038/s41418-023-01220-2 (PMC10589262; doi:10.1038/s41418-023-01220-2)

Full unedited blots for Figure 1

Figure 1E

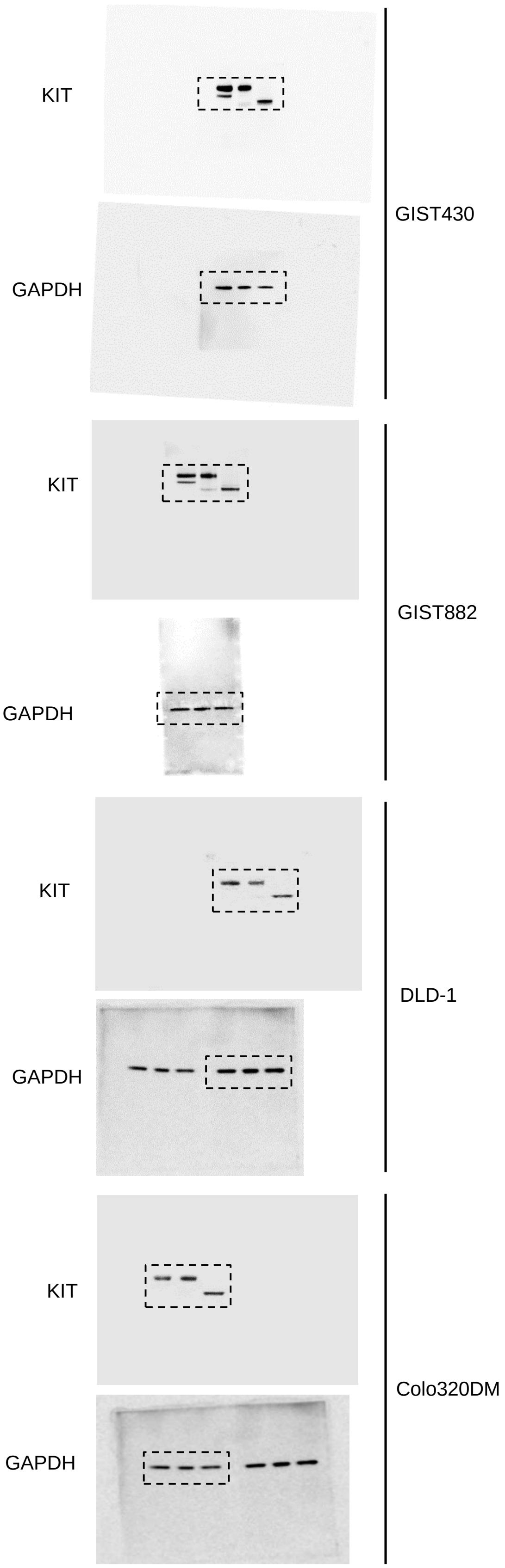

Figure 1F

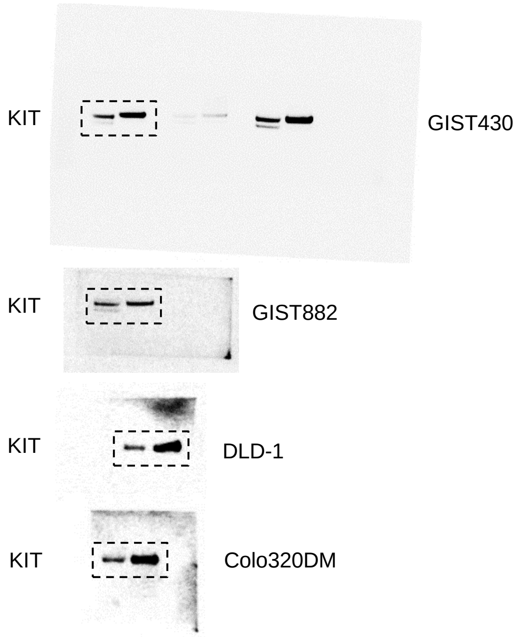

Figure 1H

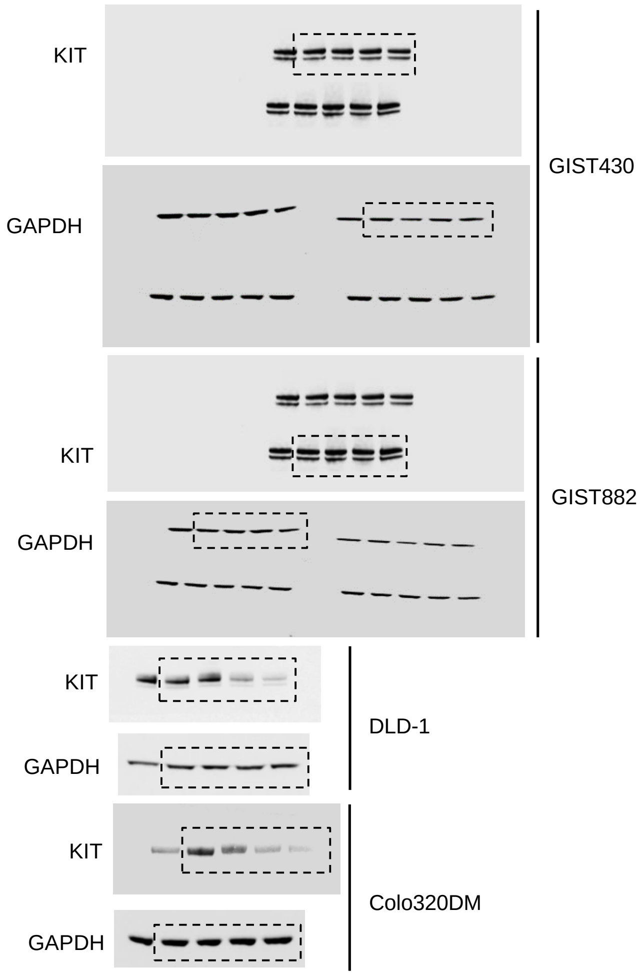

Full unedited blots for Figure 2

Figure 2A

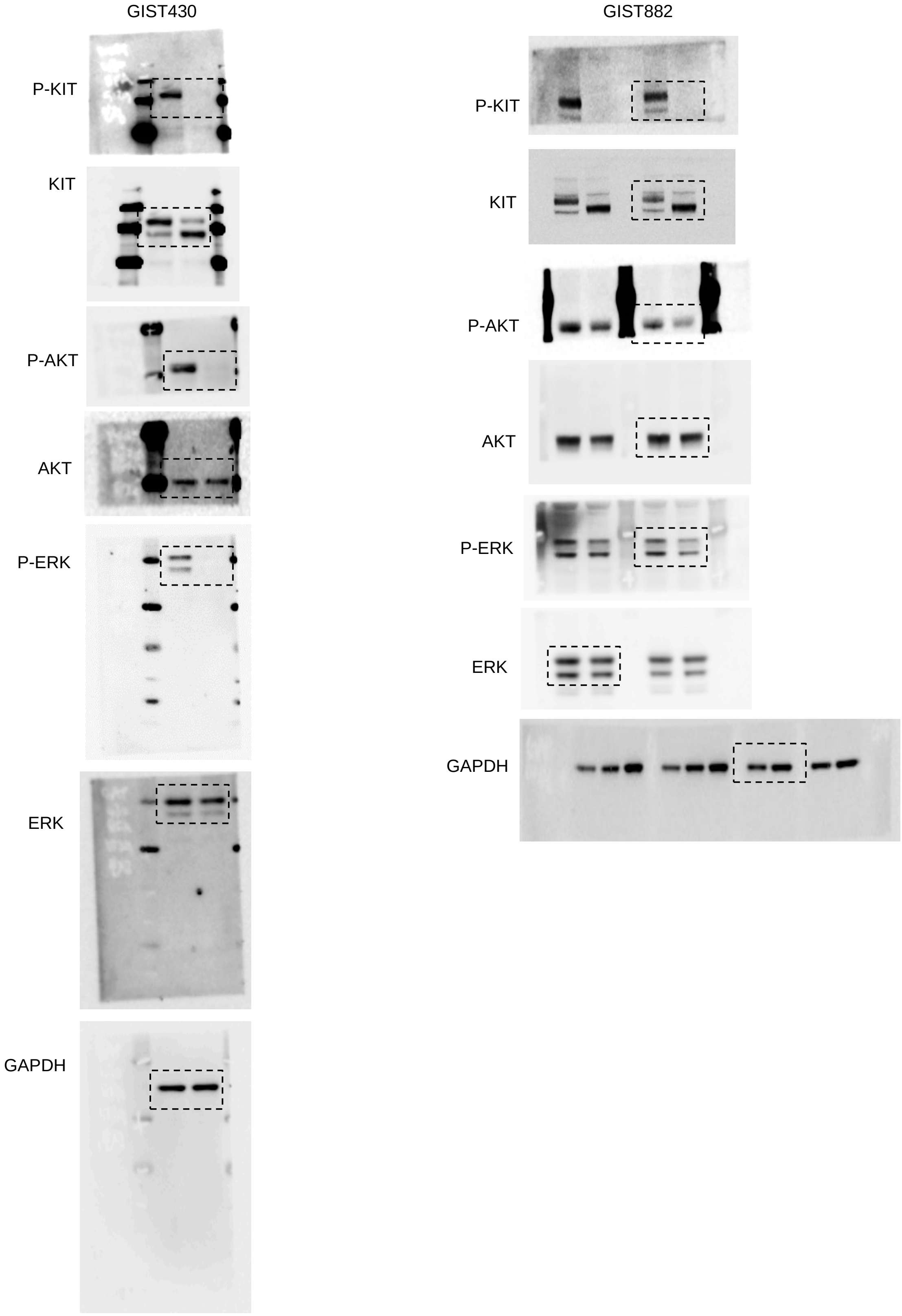

Full unedited blots for Figure 2

Figure 2B

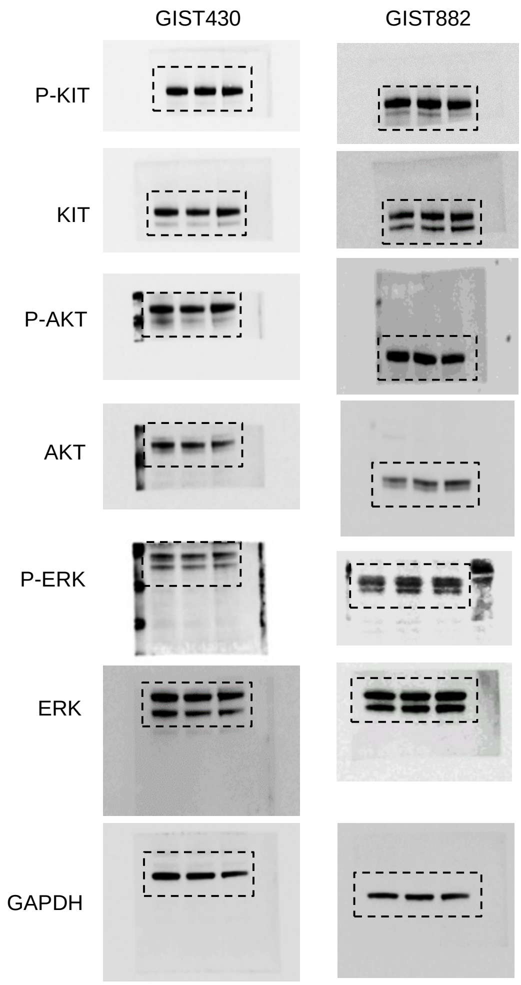

Figure 2C

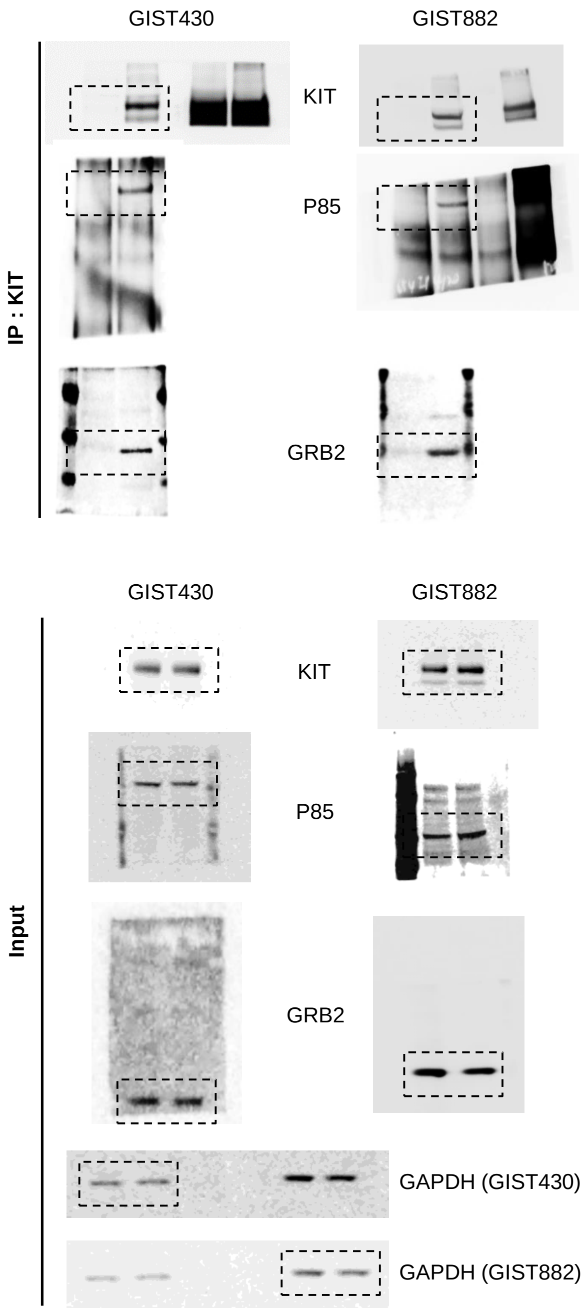

Full unedited blots for Figure 3

Figure 3A

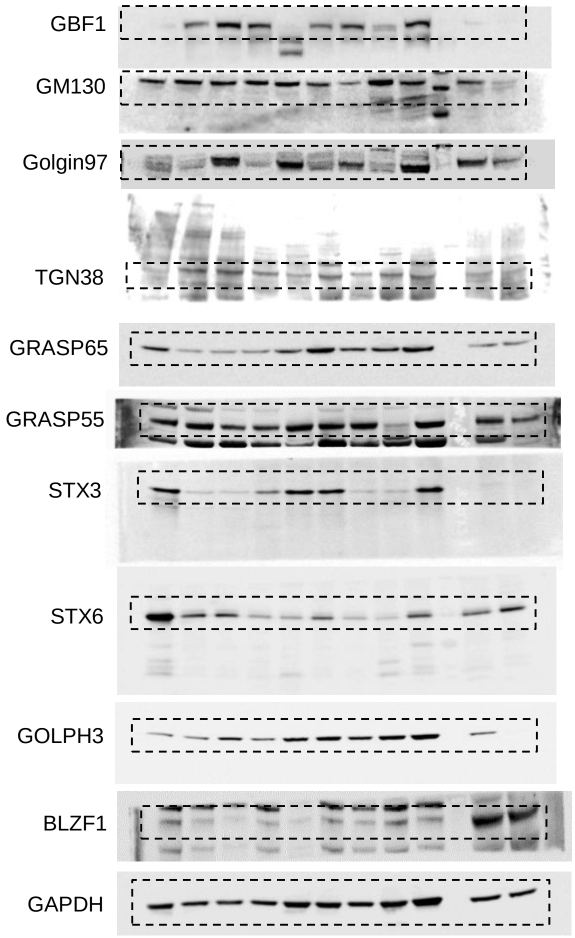

Figure 3E

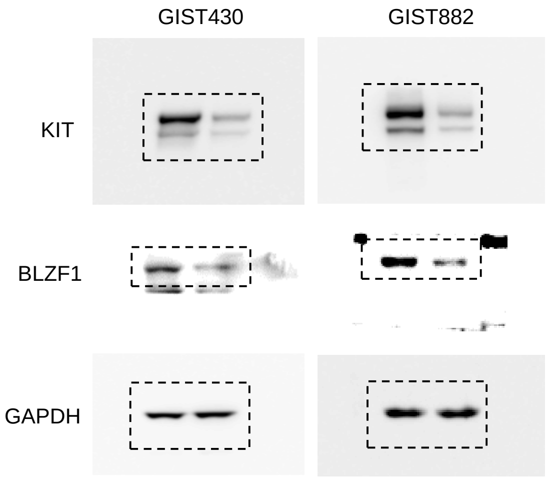

Full unedited blots for Figure 3

Figure 3B

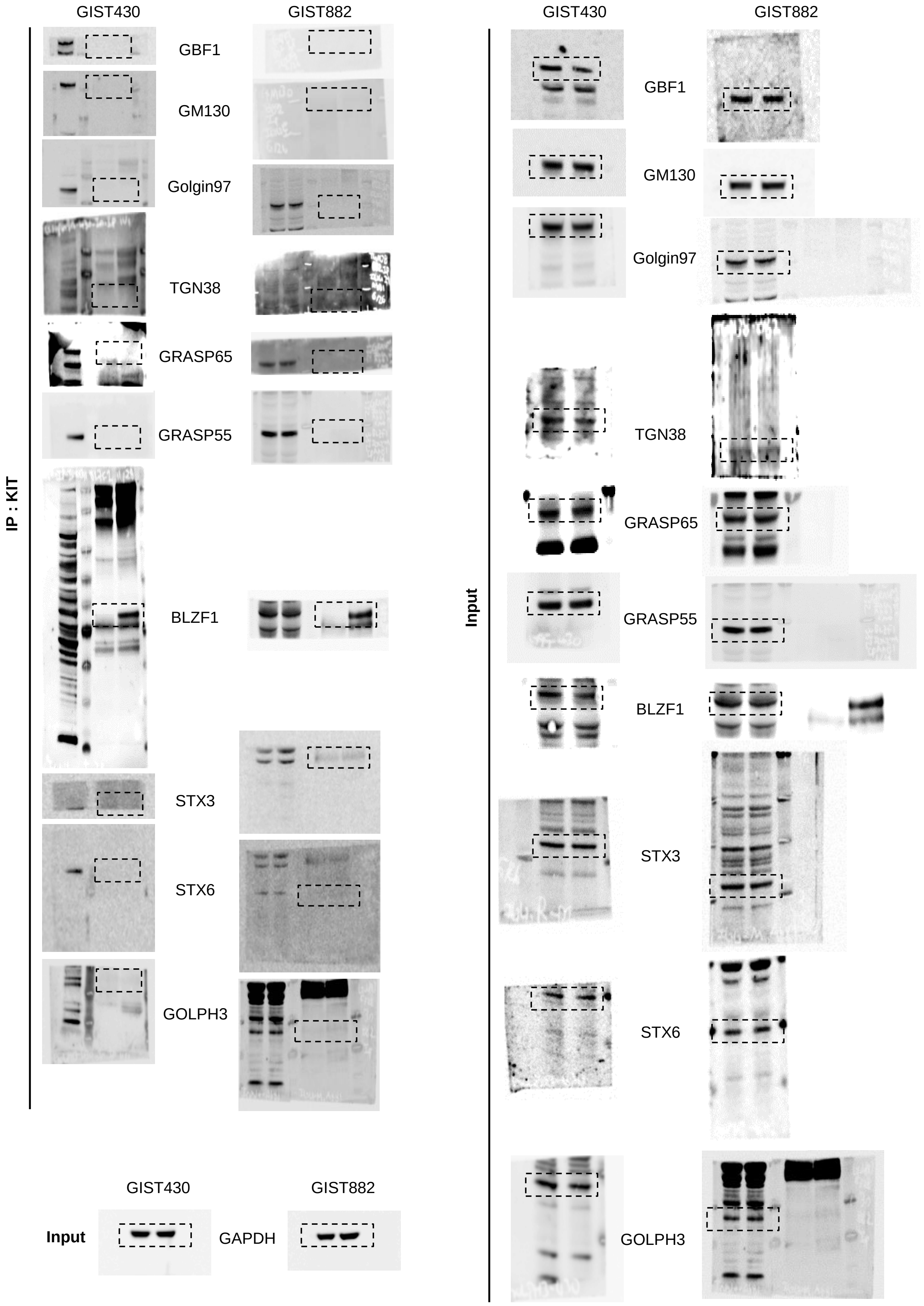

Full unedited blots for Figure 4

Figure 4A

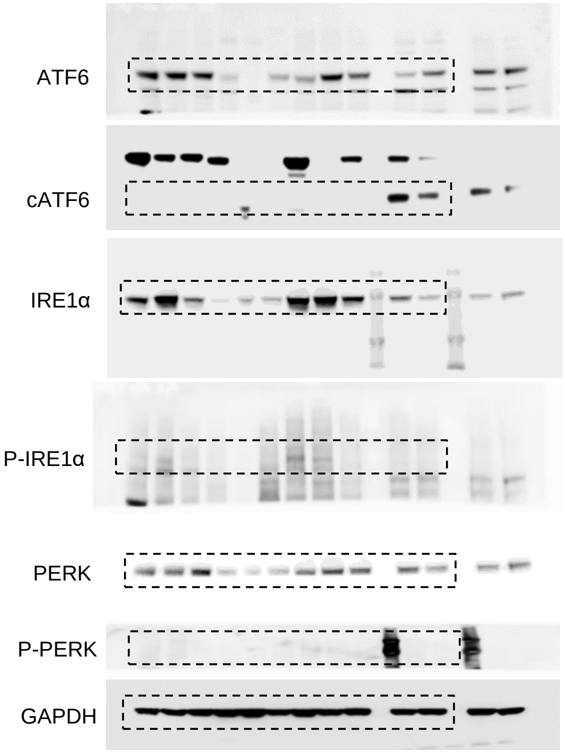

Figure 4D

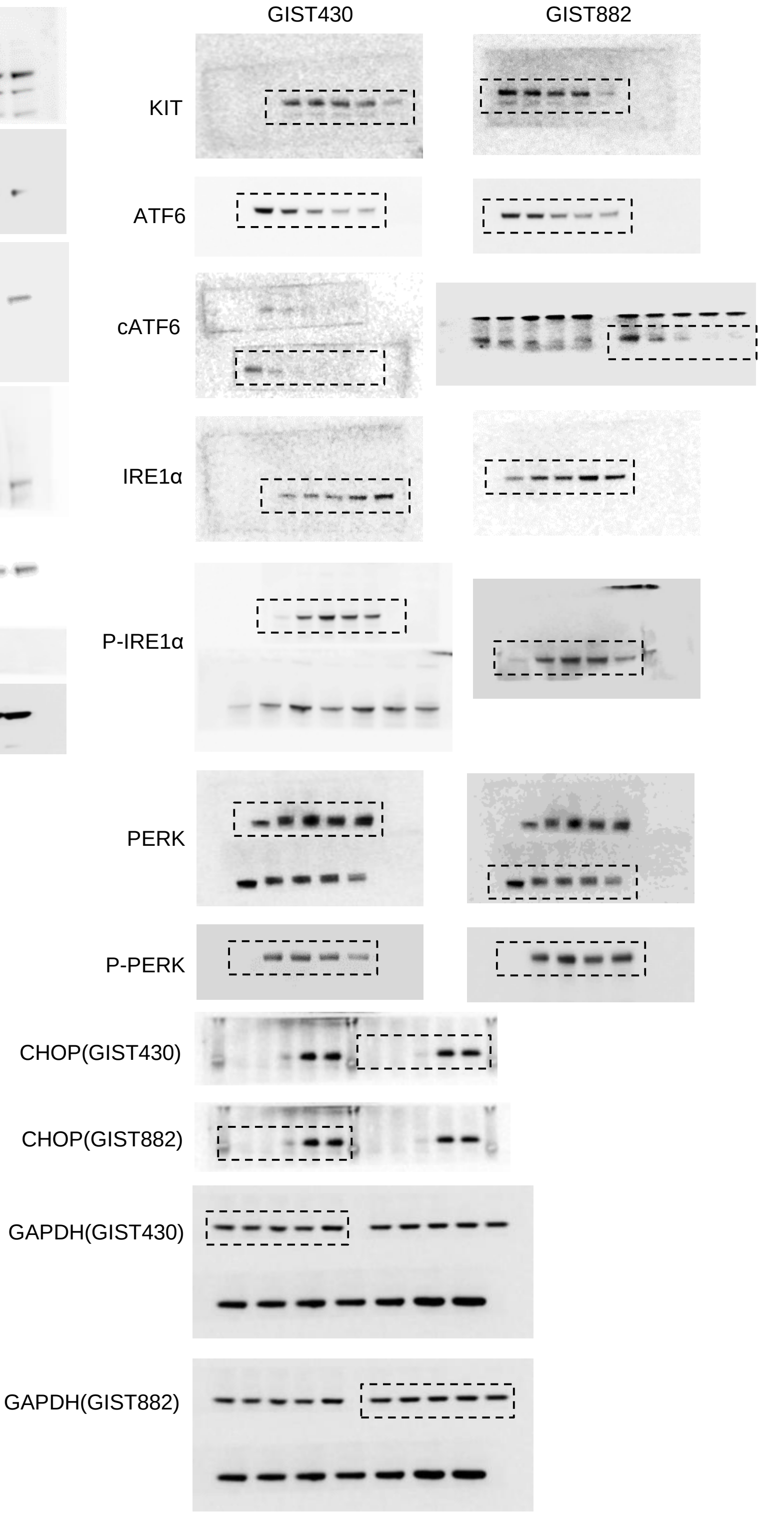

Full unedited blots for Figure 4

Figure 4E

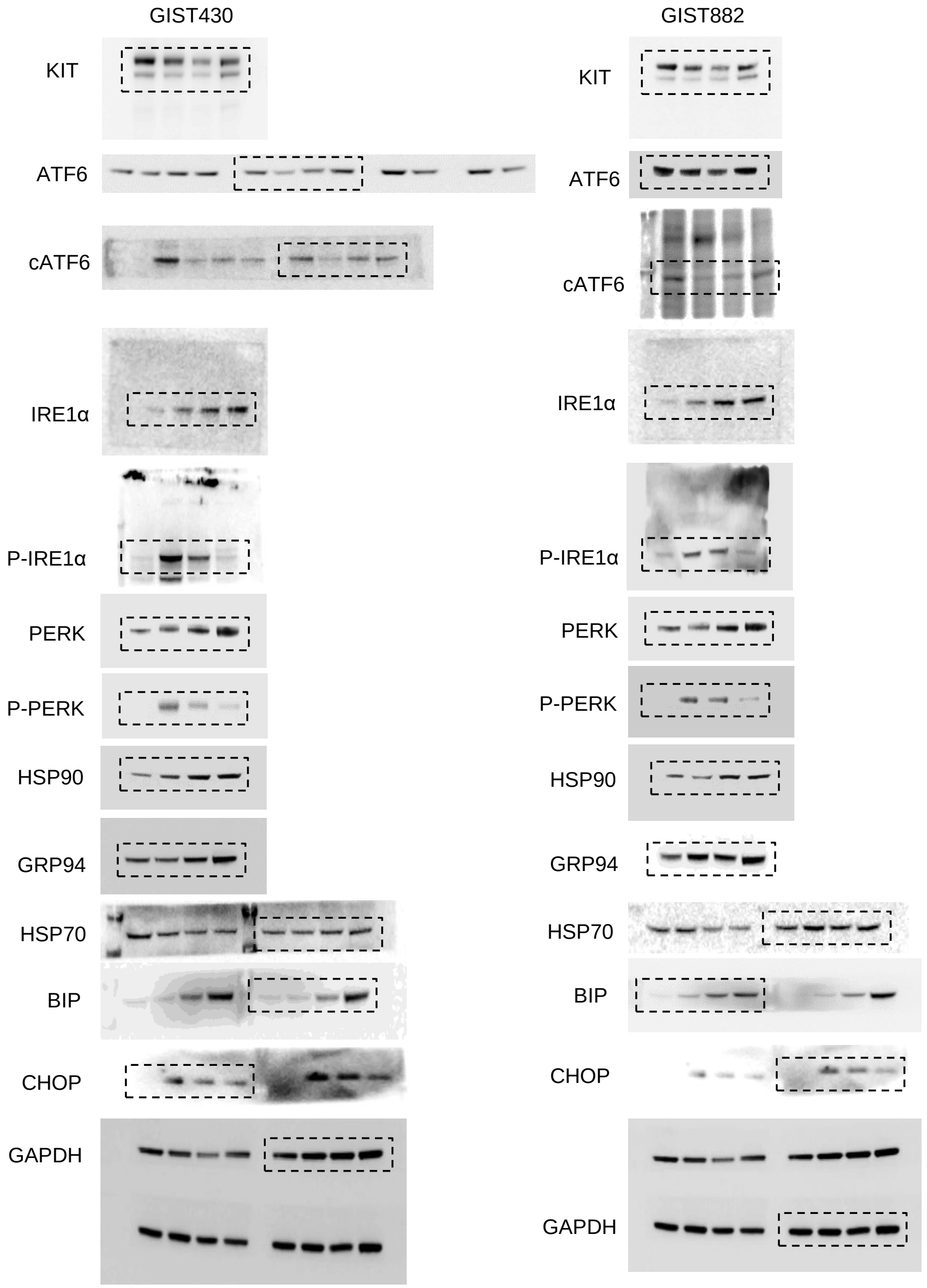

Full unedited blots for Figure 4

Figure 4F

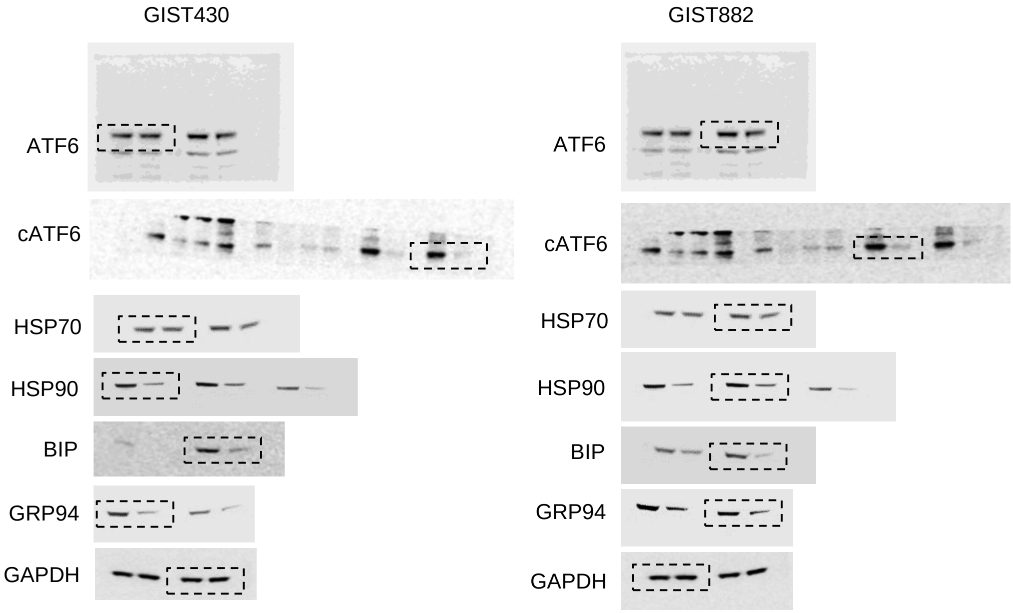

Figure 4H

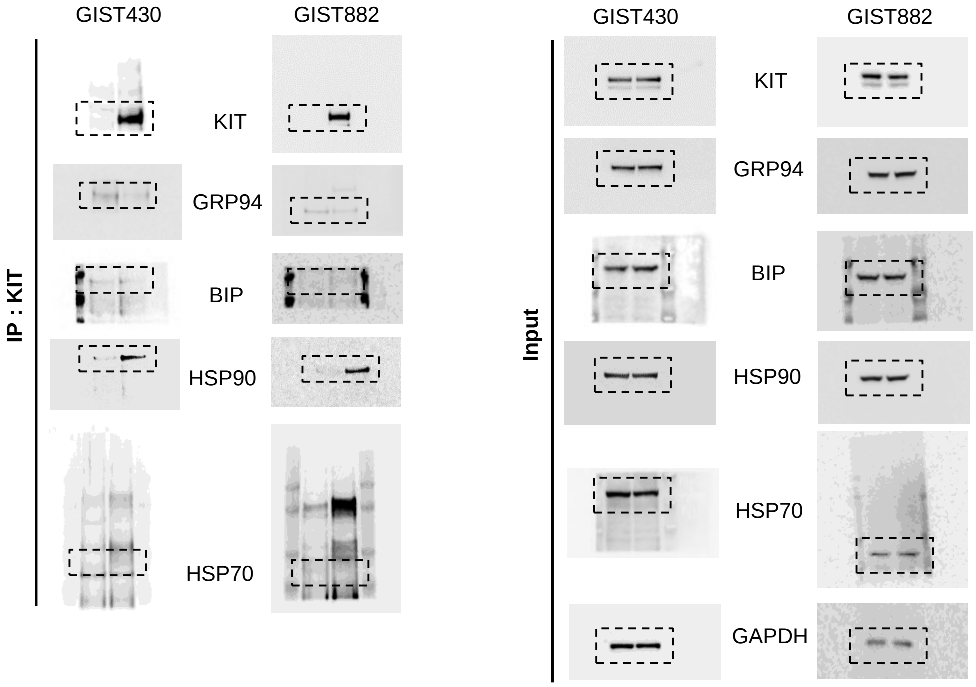

Full unedited blots for Figure 5C

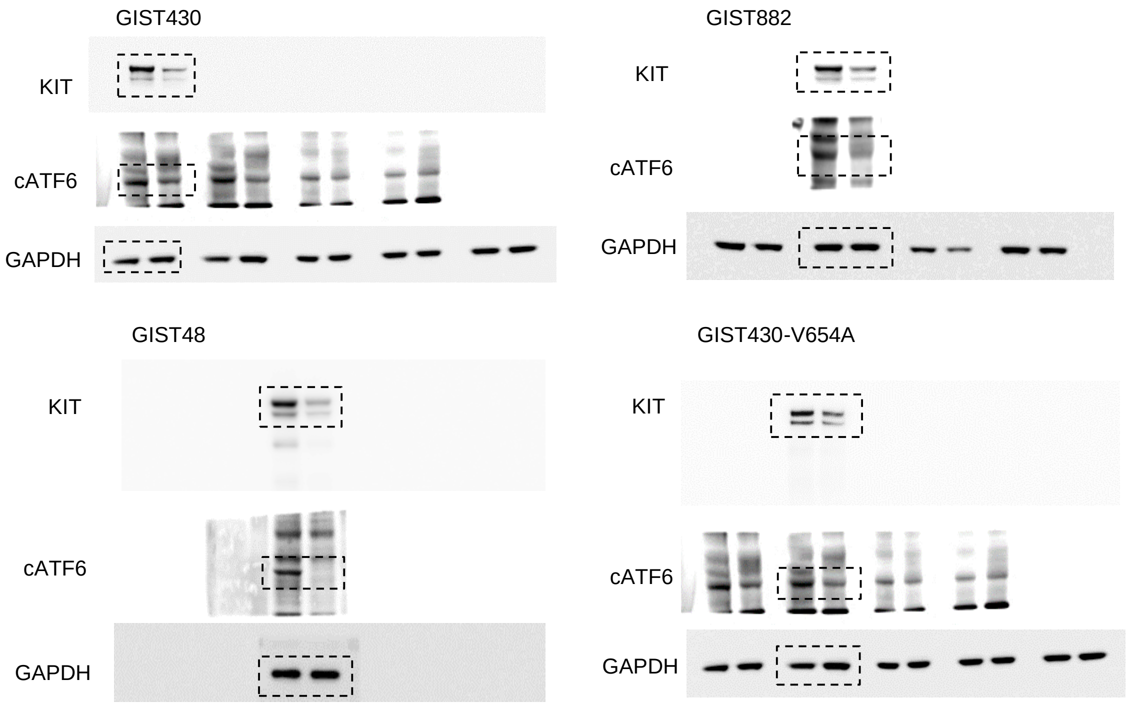

Full unedited blots for Supplementary Figure S4A

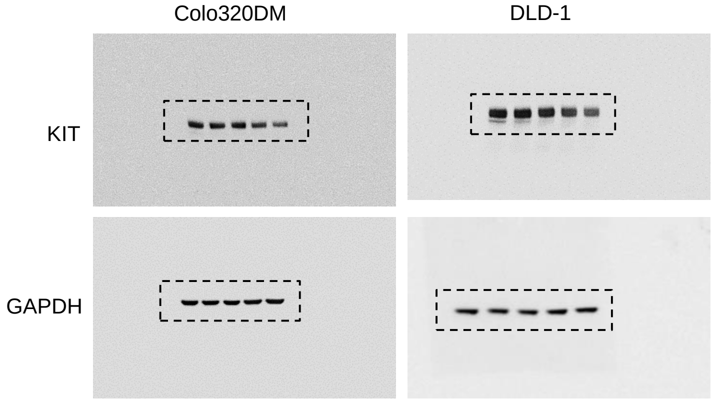

Full unedited blots for Supplementary Figure S4B

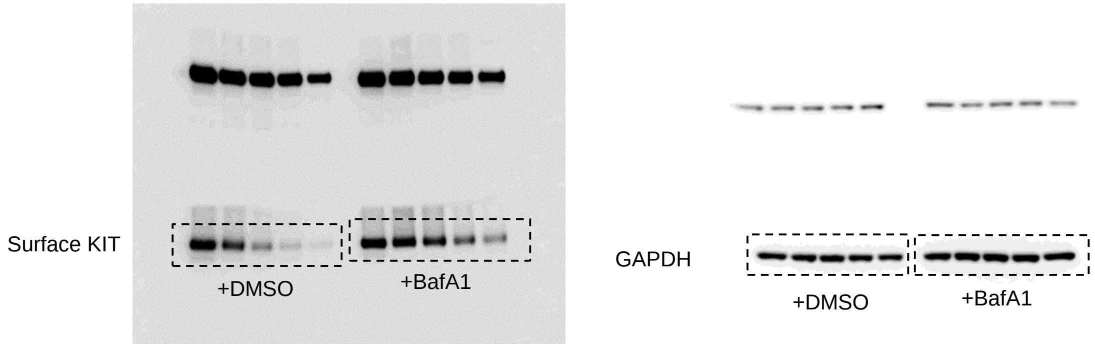

Full unedited blots for Supplementary Figure S6A

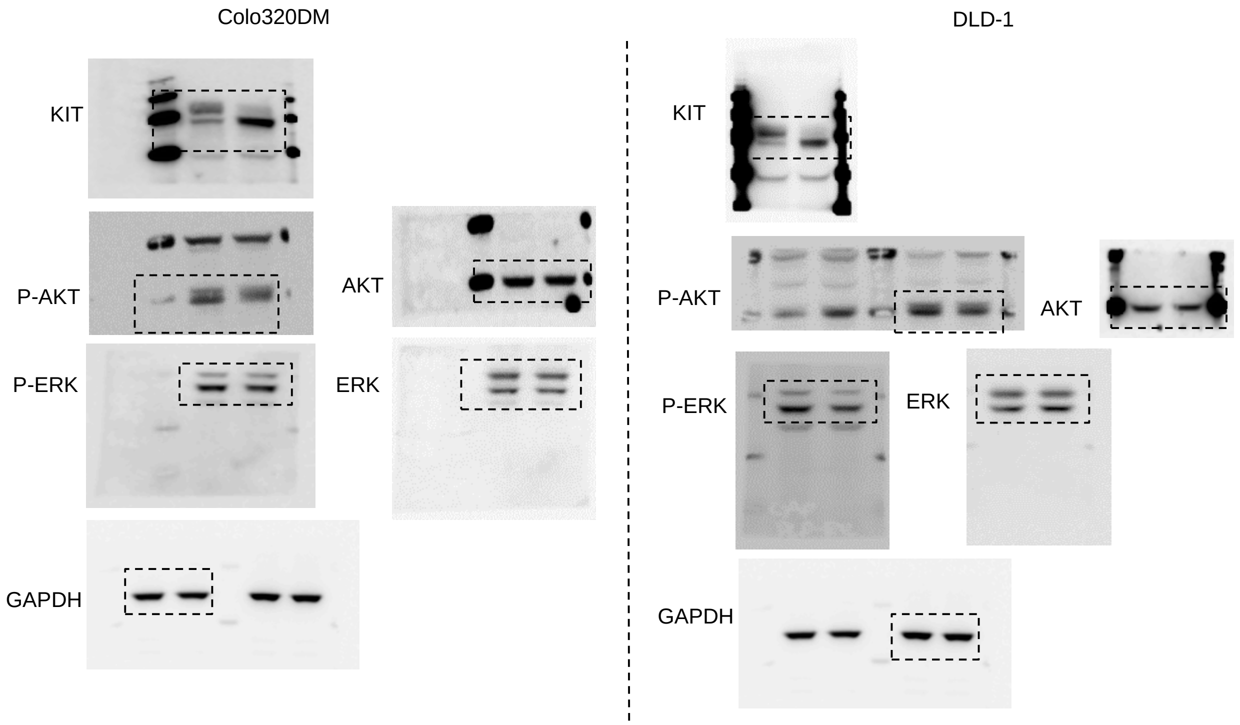

Full unedited blots for Supplementary Figure S6B

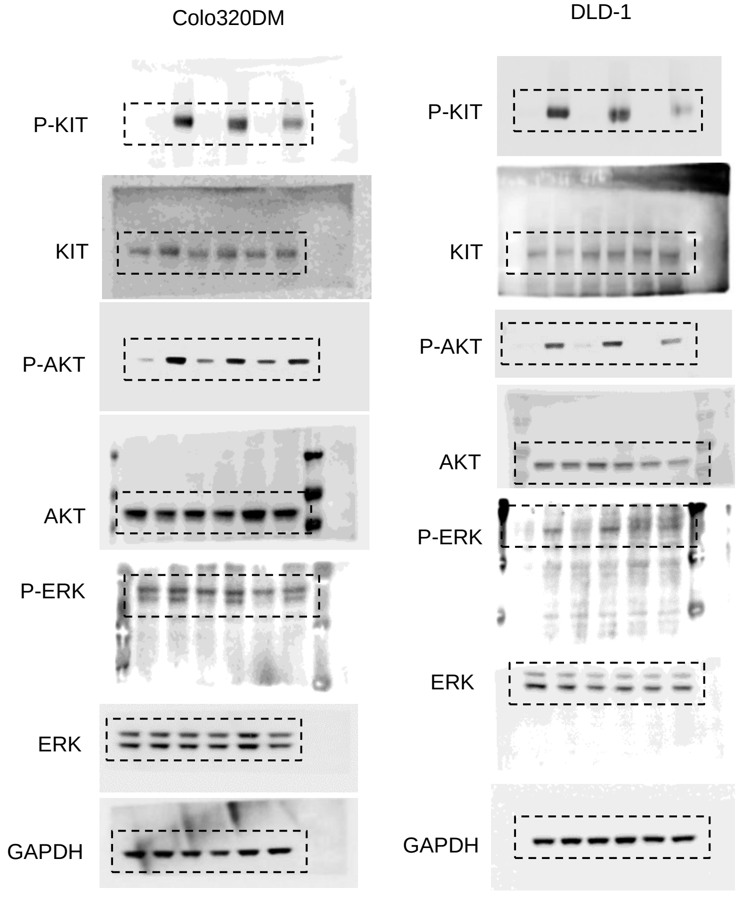

Full unedited blots for Supplementary Figure S9

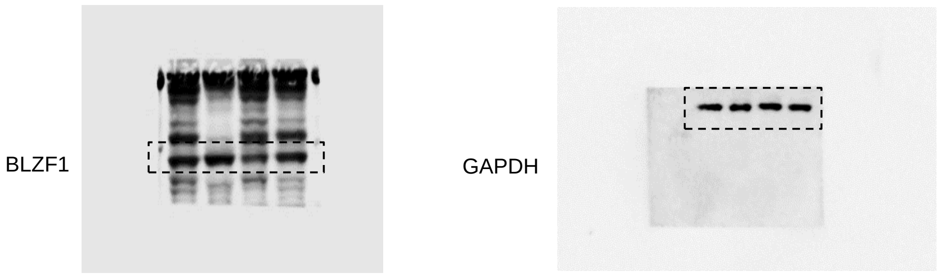

Full unedited blots for Supplementary Figure S11A

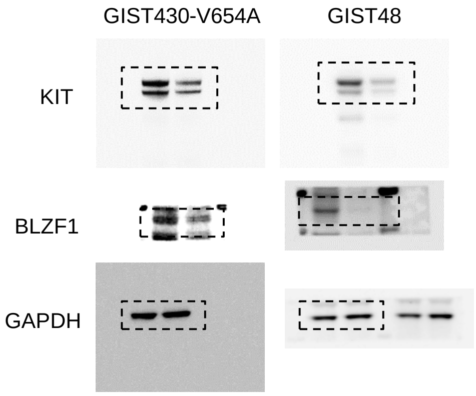

Full unedited blots for Supplementary Figure S12

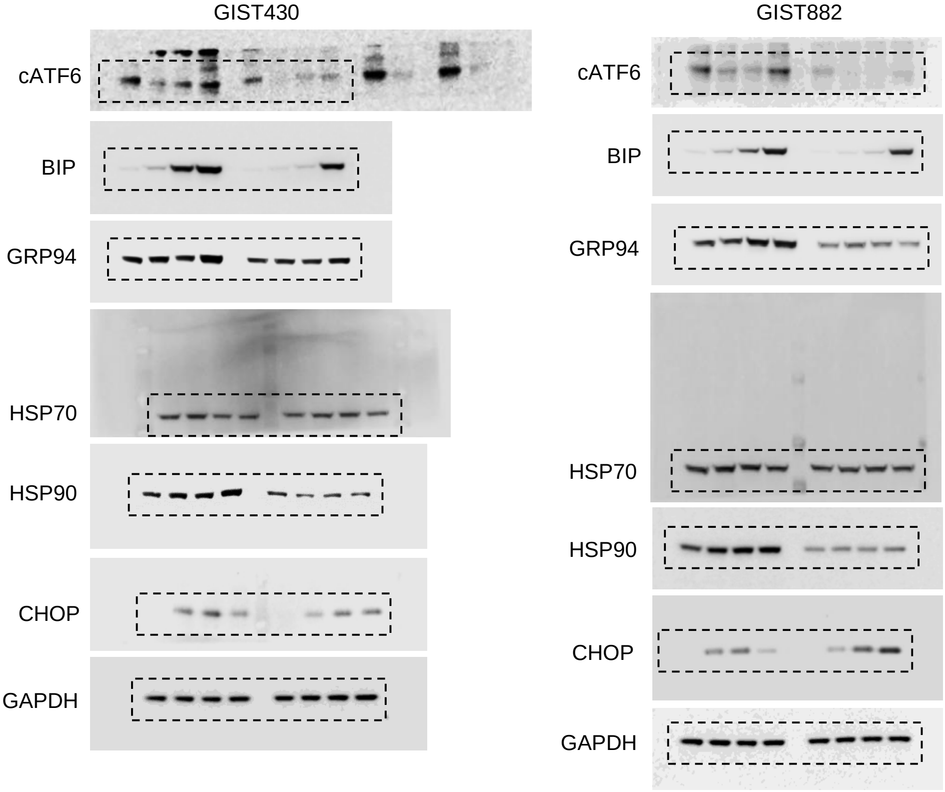

Full unedited blots for Supplementary Figure S15

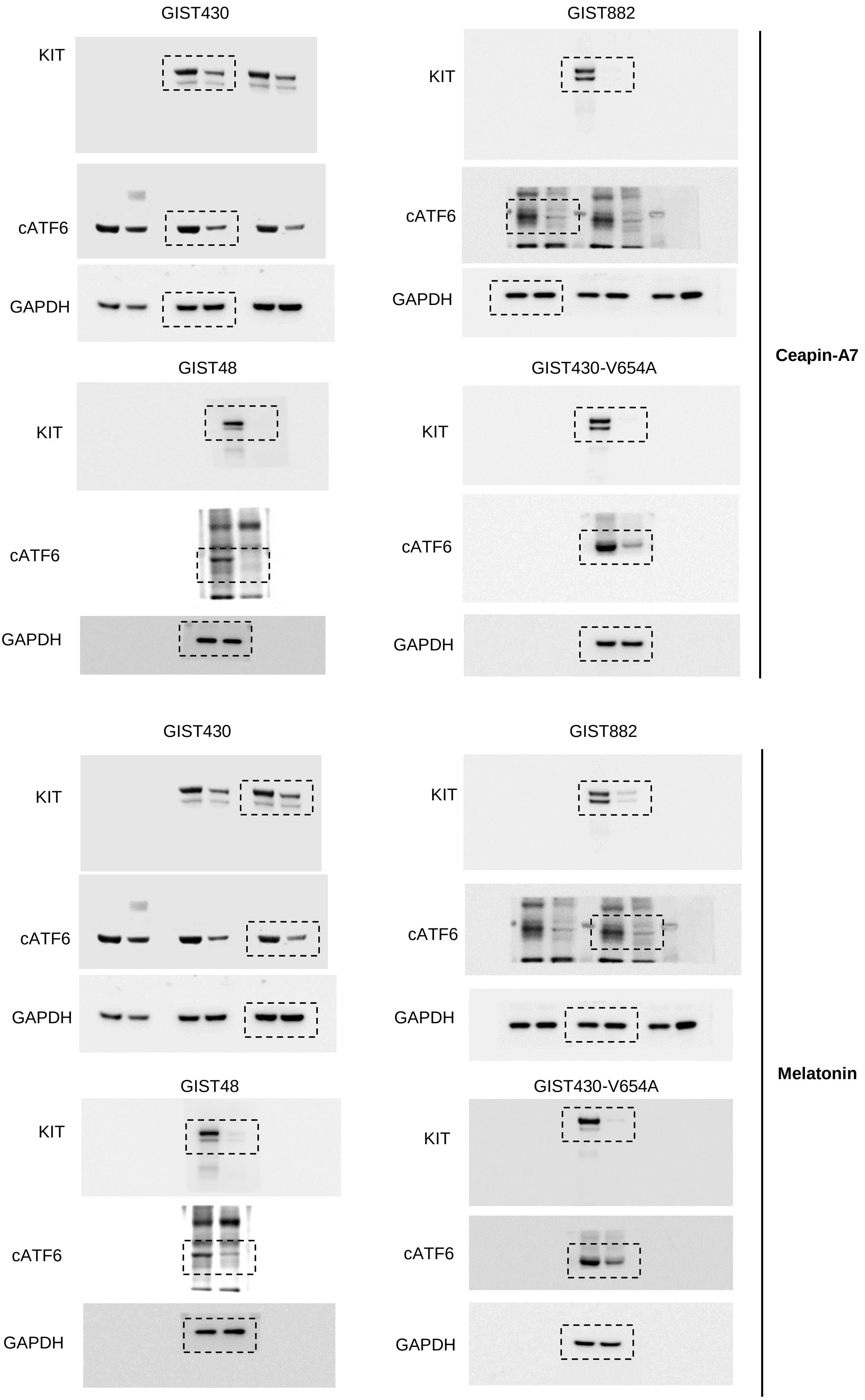

Supplement: Supplementary file 2 — Original Data File [file 41418_2023_1220_MOESM2_ESM.pdf]
