# Supplementary material for: Identification of novel pathogenic roles of BLZF1/ATF6 in tumorigenesis of gastrointestinal stromal tumor showing Golgi-localized mutant KIT
Source: Cell Death Differ. 2023 Sep 13;30(10):2309–21. doi: 10.1038/s41418-023-01220-2 (PMC10589262; doi:10.1038/s41418-023-01220-2)
Supplement: Supplementary file 3 — Reproducibility checklist [file 41418_2023_1220_MOESM3_ESM.pdf]

Corresponding Author Name: Won Kyu Kim
Manuscript Number: CDD-23-0579R

SPRINGER NATURE

Reporting Summary

Springer Nature wishes to improve the reproducibility of the work that we publish. This checklist is used to ensure good reporting standards and to improve the reproducibility. Please respond completely to all questions relevant to your man uscript. For more information, please read the journal's Guide to Authors.

☑ **Check here to confirm that the following information is available in the Material & Methods section:**

- **The exact sample size (*n*)** for each experimental group/condition, given as a number, not a range
- **A description of the sample collection** allowing the reader to understand whether the samples represent technical or biological replicates (including how many animals, litters, culture, etc.)
- **A statement of how many times the experiment shown was replicated in the laboratory**
- **Definitions of statistical methods and measures**: For small sample sizes (n<5) descriptive statistics are not appropriate, instead plot individual data points
  - Very common tests, such as *t*-test, simple χ^2 tests, Wilcoxon and Mann-Whitney tests, can be una mbiguously identified by name only, but more complex techniques should be described in the me thods section
  - Are tests one-sided or two-sided?
  - Are there adjustments for multiple comparisons?
  - **Statistical test results**, e.g., ***P* values**
  - Definition of **'center values'** as **median or mean**;
  - Definition of **error bars as s.d. or s.e.m. or c.i.**

Please ensure that the answers to the following questions are reported in the manuscript itself. We encourage you to include a specific subsection in the methods section for statistics, reagents and animal models. Below, provide the pa ge number or section and paragraph number.

Statistics and general methods

	**Reported in section/paragraph or page #**
1. How was the sample size chosen to ensure adequate power to detect a pre-specified effect size? (Give section/paragraph or page #)	Materials and methods-page #20, #21, and #23
For animal studies, include a statement about sample size estimate even if no statistical methods were used.	Materials and methods-page #20 and #23
2. Describe inclusion/exclusion criteria if samples or animals were excluded from the analysis. Were t he criteria pre-established? (Give section/paragra ph or page #)	NA
3. If a method of randomization was used to deter mine how samples/animals were allocated to ex perimental groups and processed, describe it. (G ive section/paragraph or page #)	NA
For animal studies, include a statement about ra ndomization even if no randomization was u sed.	NA

4. If the investigator was blinded to the group all ocation during the experiment and/or when as sessing the outcome, state the extent of blindi ng. (Give section/paragraph or page #)	NA
For animal studies, include a statement about blinding even if no blinding was done.	NA
5. For every figure, are statistical tests justified as appropriate?	Materials and methods-page #24
Do the data meet the assumptions of the tests (e.g., normal distribution)?	Materials and methods-page #24
Is there an estimate of variation within each group of data?	Materials and methods-page #24
Is the variance similar between the groups that are being statistically compared? (Give section/par agraph or page #)	Materials and methods-page #24

Reagents

Reported in section/paragraph or page #

6. Report the source of antibodies (vendor and catalog number)	Materials and methods-page #19 and #20
7. Identify the source of cell lines and report if they were recently authenticated (e.g., by STR profilin g) and tested for mycoplasma contamination	Materials and methods-page #18

Animal Models

Reported in section/paragraph or page #

8. Report species, strain, sex and age of animals	Materials and methods-page #23
9. For experiments involving live vertebrates, incl ude a statement of compliance with ethical reg ulations and identify the committee(s) approvi ng the experiments.	Materials and methods-page #23

10. We recommend consulting the ARRIVE guidelines (*PLoS Biol.* **8**(6), e1000412,2010) to ensure that other relevant aspects of animal studies are adequately reported.

Human subjects

Reported in section/paragraph or page #

11. Identify the committee(s) approving the study protocol.	Materials and methods-page #20, #21
12. Include a statement confirming that informed consent was obtained from all subjects.	Materials and methods-page #20, #21
13. For publication of patient photos, include a st atement confirming that consent to publish w as obtained.	NA
14. Report the clinical trial registration number (at ClinicalTrials.gov or equivalent).	NA

15. For phase II and III randomized controlled trials, please refer to the CONSORT statement and submit the CONSORT checklist with your submission.

16. For tumor marker prognostic studies, we recommend that you follow the REMARK reporting guidelines.

Data deposition

Reported in section/paragraph or page #

17. Provide accession codes for deposited data. Data deposition in a public repository is ma ndatory for:<br>a. Protein, DNA and RNA sequences<br>b. Macromolecular structures<br>c. Crystallographic data for small molecules<br>d. Microarray data	NA

Deposition is strongly recommended for many other datasets for which structured public repositories exist; more details on our data policy are available in the Guide to Authors. We encourage the provision of other source data i n supplementary information or in unstructured repositories such as Figshare and Dryad. We encourage publicati on of Data Descriptors (see Scientific Data) to maximize data reuse.

18. If computer code was used to generate results t hat are central to the paper's conclusions, incl ude a statement in the Methods section unde r "**Code availability**" to indicate whether and how the code can be accessed. Include versio n information as necessary and any restriction s on availability.	NA
